# Vaccination inducing durable and robust antigen-specific Th1/Th17 immune responses contributes to prophylactic protection against *Mycobacterium avium* infection but is ineffective as an adjunct to antibiotic treatment in chronic disease

**DOI:** 10.1080/21505594.2022.2068489

**Published:** 2022-05-01

**Authors:** Ju Mi Lee, Jiyun Park, Steven G Reed, Rhea N Coler, Jung Joo Hong, Lee-Han Kim, Wonsik Lee, Kee Woong Kwon, Sung Jae Shin

**Affiliations:** a Department of Microbiology, Institute for Immunology and Immunological Disease, Graduate School of Medical science, Brain Korea 21 Project, Yonsei University College of Medicine, Seoul, South Korea; b HDT Bio Corp, Seattle, WA, USA; c Seattle Children’s Research Institute, Center for Global Infectious Disease Research, Seattle, WA, USA; d Department of Global Health, University of Washington, Seattle, WA, USA; e Department of Pediatrics, University of Washington School of Medicine, Seattle, WA, USA; f National Primate Research Center, Korea Research Institute of Bioscience and Biotechnology, Cheongju, South Korea; g School of Pharmacy, Sungkyunkwan University, Suwon, South Korea

**Keywords:** *Mycobacterium avium* complex, Immunogenicity, Subunit vaccine, Preventative vaccine, Therapeutic vaccine

## Abstract

*Mycobacterium avium* complex (MAC) causing pulmonary disease in humanshas emerged worldwide. Thus, effective strategies simultaneously aiming to prevent MAC infection and accelerate therapeutic efficacy are required. To this end, subunit vaccine-induced protection against a well-defined virulent *Mycobacterium avium* (Mav) isolate was assessed as a preventative and therapeutic modality in murine models. Mav-derived culture filtrate antigen (CFA) was used as a vaccine antigen with glucopyranosyl lipid A stable emulsion (GLA-SE) or GLA-SE plus cyclic-di-GMP (GLA-SE/CDG), and we compared the immunogenicities, protective efficacies and immune correlates. Interestingly, CFA+GLA-SE/CDG immunization induced greater CFA-specific Th1/Th17 responses in both the lung and spleen than among the tested groups. Consequently, protective efficacy was optimally achieved with CFA+GLA-SE/CDG by significantly reducing bacterial loads along with long-lasting maintenance of antigen-specific Th1/Th17 cytokine-producing multifunctional T cell responses and relevant cytokine productions. Thus, we employed this subunit vaccine as an adjunct to antibiotic treatment. However, this vaccine was ineffective in further reducing bacterial loads. Collectively, our study demonstrates that strong Mav CFA-specific Th1/Th17 responses are critical for preventative protection against Mav infection but may be ineffective or even detrimental in an established and progressive chronic disease, indicating that different approaches to combating Mav infection are necessary according to vaccination purposes.

## Introduction

Nontuberculous mycobacteria (NTM), ubiquitous and opportunistic pathogens, include all members of *Mycobacterium* except for the *Mycobacterium tuberculosis* complex and *Mycobacterium leprae* [1,2]. Since NTM species are opportunistic pathogens, NTM infection is not mandatorily reported, unlike Mtb infection [3]. Therefore, NTM disease is difficult to diagnose, and reports of its incidence and prevalence are limited [4]. Nevertheless, the prevalence and incidence of NTM disease in humans have become a global public health problem in recent decades [2]. Ninety percent of NTM infections cause pulmonary disease (PD) [5]. Representatively, the prevalence of NTM-PD was approximately 13.9/100,000 patients in the USA in 2013, 36.1/100,000 patients in South Korea in 2016, 24.9/100,000 patients in Japan in 2016, 46.0/100,000 patients in Taiwan in 2014, and 6.2/100,000 patients in five EU countries (United Kingdom, France, Germany, Italy, and Spain) in 2016 [5–10]. Although the distribution of NTMs causing PD geographically differs, the *Mycobacterium avium* complex (MAC), mainly consisting of *M. avium* subspecies *hominissuis* (hereafter referred to as Mav) and *M. intracellulare*, is considered the main causative agent among NTM species worldwide [2,11].

MAC-induced PD (MAC-PD) patients usually receive at least three antibiotics, macrolides, rifampicin (RIF), and ethambutol, as a recommended standard regimen for at least one year after reaching negative culture conversion [12,13]. Indeed, a decrease in the bacterial burden within the first few months after treatment is a major predictable marker of maintenance of long-term sputum conversion status [14]. Therefore, patients are subsequently monitored for therapeutic success and failure every 1–2 months of the treatment duration [15]. Nevertheless, the treatment outcome of patients with MAC-PD remains disappointing, and the lower therapeutic success rate (68.1%) may be caused by drug resistance, relapse, or recurrent infections with different NTM strains or species after completion of treatment [2,16–19]. To date, a treatment method after therapeutic failure is unavailable, and mortality is subsequently increased [20,21]. More than 25% of the high mortality rate was reported during a 5-year follow-up [19,22]. Although few studies have described the treatment outcomes and clinical characteristics of patients who had acquired macrolide resistance or treatment failure without any mutation (referred as the refractory MAC-PD), the continuous prescription of macrolide is used for those patients because of the limited availability of alternative drugs [17,20,21,23,24]. Thus, improved preventive and therapeutic interventions against MAC infection are considered a prompt unmet need.

Recently, Larsen *et al*. reported a proof-of-concept study using ID91, which contains Mav antigen as well as Mtb, adjuvanted in glucopyranosyl lipid A stable emulsion (GLA-SE), a well-defined Th1-biasing Toll-like receptor 4 adjuvant, and this type of subunit vaccine exhibited protective efficacy against Mav infection in two different mouse strains with different susceptibilities: C57BL/6 and immunocompromised Beige mice. Importantly, significant reductions in pulmonary bacterial loads were generated by vaccination with ID91/GLA-SE or bacille Calmette-Guerin (BCG) in both mouse strains, indicating that prophylactic immunization may be an effective strategy against Mav infection [25].

To date, the protective immune correlates against MAC are not completely understood; however, in general, Th1-type T cell immunity may have a critical role in protection. According to previous studies, the number of inflammatory cells, nitric oxide (NO) production, and IFN-γ production were decreased in IL-12-depleted mice, resulting in increased susceptibility to Mav infection [26–28]. Similarly, disseminated Mav infections have been reported to affect individuals with Mendelian susceptibility to mycobacterial diseases who are deficient for the IL-12/IFN-γ axis, which is representative of Th1 responses [29,30]. In addition, the increases in the incidence of Mav infection caused by HIV infection and post-TNF-α blockade therapies may support the hypothesis that Th1 responses have a critical role in protecting against Mav infection [31]. Along with reduced Th1 responses that are factors for susceptibility to MAC infection, lower levels of Th17-associated cytokines have been detected in patients with MAC-PD compared to those in healthy controls [32,33]. Notably, significantly reduced production of Th1 cytokines (i.e., CD40L and IFN-γ) in patients at the time of diagnosis was rescued after treatment, while the levels of Th17-associated cytokines were further decreased at one year after treatment compared to the pretreatment levels [33]. Moreover, our group recently demonstrated that exacerbation of Mav infection is likely associated with disrupted Mav culture filtrate antigen (CFA)-specific Th17 responses when accompanied by allergic asthma [34]. Additionally, Kannan *et al*. reported that mice immunized with MPT64 (a Mav antigen)-expressing *M. smegmatis* Δ*espG*_3_ exhibited a significantly reduced Mav burden along with a large number of Mav-specific CD4^+^ and CD8^+^ T cells expressing IL-17, indicating that Th17 responses that are enhanced by Mav are also pivotal to the control of Mav infection [35].

Although the functionality of Th17 immunity in the pathogenesis of intracellular bacterial diseases, including mycobacterial diseases, is controversial, antigen-specific Th17 responses against Mtb infection are known as an essential host protection component in the field of vaccine development [36–39]. Likewise, enhancing antigen-specific Th17 responses may provide a potential strategy for the development of a vaccine against Mav infection.

Recently, Park *et* *al*. discovered the role of c-di-GMP (CDG) as an educational signal for inducing Th17 cell differentiation via the stimulator of interferon genes (STING) pathway. STING activation by CDG prompted the migration of mucosal dendritic cells (DCs) with IRF1-dependent transcriptional programs and significantly increased the frequencies of Th17 cells in mesenteric lymph nodes. Thus, the STING-IRF1 signaling pathway was shown to be crucial to the initiation of mucosal Th17 responses, thereby activating host defenses against invasive intracellular pathogens such as *Salmonella Typhimurium* [40]. In addition, STING-activating cyclic dinucleotides (CDNs) both elicited Th1 and Th17 responses and protected against Mtb infection [41].

In the past decade, therapeutic vaccines preventing NTM infection recurrence as well as tuberculosis (TB) have received attention in the mycobacterial vaccine field as a major advancement in the development of chemotherapies with significantly enhanced efficacies and shorter treatment regimens [42]. However, the therapeutic vaccine has been proven to be effective for TB but has not been proven for NTM disease. Coler *et* *al*. demonstrated that compared with antibiotic treatment alone, therapeutic immunization in combination with antibiotic treatment (RIF and isoniazid) induced robust and durable multifunctional antigen-specific Th1 responses, thereby reducing the bacterial burden, conventional chemotherapeutic treatment duration and Mtb-induced pulmonary inflammation [42]. In addition, RIF, isoniazid, and pyrazinamide treatment with ID93+GLA-SE was determined to have protective efficacy and to relatively enhance the levels of Th1-associated cytokines [43]. However, antibiotic treatment alters immune responses [44,45], and therapeutic vaccination strategies for MAC infection in combination with antibiotic treatment may differ from those utilized for Mtb infection. Therefore, we attempted to induce antigen-specific Th1/Th17 responses via therapeutic immunization with Mav-derived CFA adjuvanted with GLA-SE plus CDG in combination with antibiotic treatment for the first time.

For the reasons stated above, we herein compared the effects of vaccines developed using CFA as an antigen that were differentially adjuvanted with GLA-SE or GLA-SE plus CDG against a well-defined virulent Mav clinical isolate, as a preventative and therapeutic strategy, in a murine model of chronic progressive disease. In addition, we investigated the qualitative and quantitative correlates of protection against chronic progressive MAC infection following immunization with preventative and therapeutic subunit vaccine regimens.

## Materials and methods

### Ethics statement

Animal experiments were carried out according to the instructions provided by the Korean Food and Drug Administration. All protocols and procedures were approved by the Ethics Committee and Institutional Animal Care and Use Committee (permit numbers: 2018-0229 and 2020-0083) at Yonsei University College of Medicine (Seoul, South Korea).

### Mycobacterial strain, culture, and antigen preparation

The macrolide-susceptible (MIC = 0.5 µg/ml) virulent Mav SMC #7, clinically isolated from a typical MAC-PD patient, was used throughout the current study to generate a chronically progressive Mav infection model as previously described [46]. Briefly, Mav SMC #7 cells were cultured in Middlebrook 7H9 broth (BD-Difco, Le Pont de Claix, France) containing 10% oleic acid-albumin-dextrose-catalase (OADC) at 37°C collected, and stored at −80°C until use, as previously described [46]. For infection experiments, the number of colony-forming units (CFUs) was predetermined by plating the stock on Middlebrook 7H10 agar (BD-Difco). For CFA preparation, Mav SMC #7 was grown in modified Watson-Reid (mWR) (pH 6.0) broth for 6 weeks at 37°C to harvest and pool the antigen as previously described [47]. The mWR broth, which is the protein-free medium, was recommended for use in the preparation of mycobacterial lysate and culture filtrate proteins in previous studies [47–49]. Mav SMC #7 in mWR broth was centrifugated at 10,000 × g for 30 min, and Mav SMC #7 was filtered using a 0.2-μm-pore-size filter (Nalge Nunc International, Rochester, NY, USA). Then, the filtrate was collected by ultrafiltration using a Centricon Plus-80 device (molecular weight cutoff, 5 kDa; Merck, Darmstadt, Germany) and dialyzed five times against phosphate-buffered saline (PBS; LPS Solution, Daejeon, South Korea) by a Slide-A-Lyzer dialysis cassette (Pierce Biotechnology, Rockford, IL, USA). The CFA concentration was estimated using a Bio–Rad Protein Assay Kit (Hercules, CA, USA).

### Mice, immunization, and challenge protocol

Six- to eight-week-old, specific pathogen-free, female C57BL/6 and BALB/c mice from Japan SLC, Inc. (Shizuoka, Japan) were used after adaptation for 1 week in before challenge. Mice were housed in an animal biosafety level (ABSL)-3 facility and provided water and a sterile commercial diet *ad libitum* at a consistent temperature (24 ± 1°C) and humidity (50 ± 5%) on a controlled light dark cycle (switched on/off every 12 h). No mice showed any observable symptoms or deterioration during daily monitoring and none of the mice were pre-determined to require sacrifice before the endpoint of this experiment.

The two strains of mice were infected with Mav SMC #7 via the aerosol route using a Glas-Col aerosol apparatus (Terre Haute, IN, USA), as previously described [46], to compare disease severity between C57BL/6 and BALB/c mice. At 10 weeks post-infection, five mice from each group were euthanized for pathological and bacterial load analyses (Supplementary Figure S1A).

**Figure 1:**
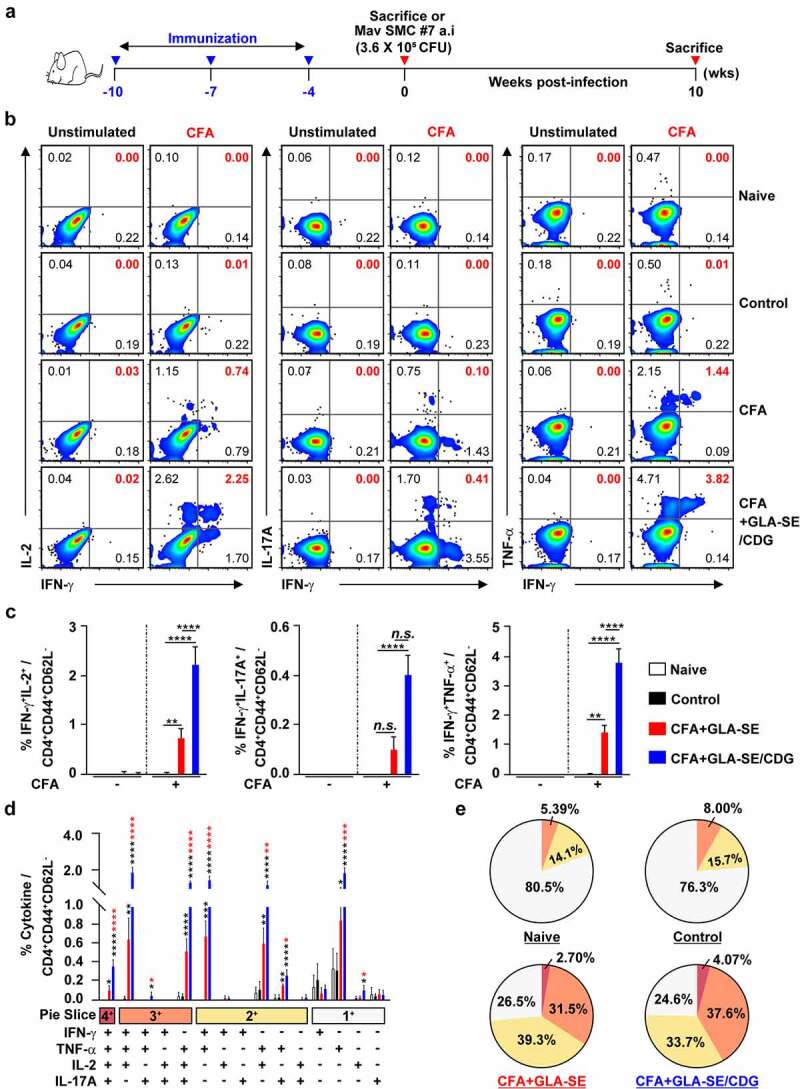
Qualitative and quantitative analyses of Mav CFA-specific multifunctional CD4^+^ T cells in the lung after CFA+GLA-SE or CFA+GLA-SE/CDG immunization. Four weeks after the last immunization, mice were sacrificed, and lung single cells harvested from each group (*n* = 4) were stimulated by GolgiPlug and GolgiStop with or without 10 µg/ml CFA at 37°C for 9 h. (a) Scheme of the *in vivo* experiment for immunogenicity and vaccine efficacy analysis. The frequencies of Mav CFA-specific IFN-γ^+^IL-2^+^-, IFN-γ^+^IL-17A^+^- and IFN-γ^+^TNF-α^+^-expressing CD4^+^CD44^+^CD62L^−^ T cells were assessed after staining of intracellular cytokines and are presented as (b) pseudocolor dot plots and (c) summary bar graphs. (d) The percentages of total CD4^+^CD44^+^CD62L^−^ T cells with differential production of IFN-γ, IL-2, IL-17A and TNF-α in response to CFA stimulation among lung single cells were determined among groups and are presented as bar graphs. (e) The values of the proportions of quadruple- (4+, crimson), triple- (3+, orange), double- (2+, yellow), and single-function (1+, light gray) CD4^+^CD44^+^CD62L^−^ T cells expressing IFN-γ, IL-2, IL-17A and TNF-α in each immunized group are illustrated as pie charts. Statistically significant differences among all groups in (c) and (d) were determined by one-way ANOVA with Tukey’s multiple comparison test, and the results are presented as the mean values along with the S.Ds. *p < .05, **p < .01, ***p < .001, ****p < .0001 and *n.s*.: not significant. The asterisks in (d) represent significant differences between groups: black, control group vs. CFA+GLA-SE/CDG group; red, CFA+GLA-SE group vs. CFA+GLA-SE/CDG group. The representative results are shown from a single *in vivo* experiment. CFA, culture filtrate antigen; Control, GLA-SE immunization alone; GLA-SE, glucopyranosyl lipid A adjuvant formulated in a stable oil-in-water emulsion; GLA-SE/CDG, GLA-SE plus cyclic-di-GMP.

For preventative vaccination, 5 µg of CFA was incorporated into 5 µg of GLA-SE (IDRI; Seattle, WA, USA), referred to as CFA+GLA-SE, and 5 µg of CFA and 5 µg of CDG (InvivoGen, San Diego, CA, USA) were incorporated into 5 µg of GLA-SE, referred to as CFA+GLA-SE/CDG. Then, each vaccine was intramuscularly administered three times at 3-week intervals (Figure 1a and Supplementary Table S1). The control group received intramuscular injection of GLA-SE alone. Four weeks after the final vaccination, the vaccinated mice were infected with Mav SMC #7 via the aerosol route. At 10 weeks post-infection, the mice were euthanized, and their lungs and spleens were homogenized for bacterial CFU, lung inflammation and relevant immunological analyses.

For therapeutic vaccination, the mice were first infected with Mav SMC #7 via the aerosol route. At 8 weeks post-infection, treatment with 100 mg/kg clarithromycin (CLR) (Sigma–Aldrich, St. Louis, MO, USA) was initiated for 8 weeks, and the mice were immunized 3 times (9, 12, and 15 weeks post-infection) with CFA+GLA-SE/CDG during CLR administration (Supplementary Table S1). At 19 weeks post-infection, therapeutic efficacy was determined based on the assessment of immune responses, bacterial loads, and pathology.

### Flow cytometry analysis of intracellular cytokine production

For preparation of single-cell suspension, each lung tissue was chopped into small pieces and incubated with Roswell Park Memorial Institute 1640 Medium (RPMI 1640; Biowest, Nuaillé, France) containing 10% fetal bovine serum (FBS; Biowest) containing 0.1% collagenase type II (Worthington-Biochem, Lakewood, NJ, USA) at 37°C for 30 min. The single-cell suspensions were then washed with RPMI 1640 containing 2% FBS and harvested after centrifugation at 2,000 rpm for 3 min. Red blood cells from lung and spleen cells were lysed using ACK lysis buffer (ThermoFisher Scientific, Waltham, MA, USA). Then, lung and spleen cells were harvested after centrifugation at 2,000 rpm for 3 min and resuspended in RPMI 1640 containing 10% FBS. Lung and spleen single-cell preparations (1 × 10^6^ cells) from a subset of groups were plated in 96-well cell culture plates and stimulated with 10 μg/ml CFA supplemented with GolgiPlug and GolgiStop (BD Biosciences, Franklin Lakes, NJ, USA). After 9 h of stimulation, the cells were washed with PBS containing 2% FBS and Fc receptors were blocked by an incubation with an anti-CD16/32 antibody (BioLegend, San Diego, CA, USA) at 4°C for 20 min. Next, the cells were then stained with LIVE/DEAD^TM^ Fixable Viability Dye eFluor^TM^ 780 (ThermoFisher Scientific), Violet (V) 450-conjugated anti-CD44 antibodies, peridinin chlorophyll (PerCP)-Cy5.5-conjugated anti-CD4 (BD Biosciences) antibodies, Brilliant Violet (BV) 605-conjugated anti-CD90.2 antibodies, BV785-conjugated anti-CD8a antibodies, and Alexa700-conjugated anti-CD62L antibodies (BioLegend) at 4°C for 30 min. Following the removal of the remaining antibodies, the cells were fixed with a Cytofix/Cytoperm Kit (BD Biosciences) at 4°C for 30 min. Ultimately, after washing the cells twice with Perm/Wash (BD Biosciences), Alexa488-conjugated anti-IL-17A, phycoerythrin (PE)-conjugated anti-IFN-γ, PE-Cy7-conjugated anti-IL-2, and allophycocyanin (APC)-conjugated anti-TNF-α antibodies (BD Biosciences) were used for intracellular staining, the cells were washed three times and then fixed with IC Fixation buffer (Invitrogen, Waltham, MA, USA). The detailed information of antibodies was summarized in Supplementary Table S2. Then, the cells in PBS containing 2% FBS were assayed on a CytoFLEX S flow cytometer (Beckman Coulter, Indianapolis, IN, USA) and analyzed using FlowJo software (Tree Star, Ashland, OR, USA) in accordance with the illustrated gating strategy (Supplementary Figure S2).

### Antibody titers in serum

The Mav CFA-specific total IgG, IgG1, IgG2a, and IgG2b levels in the serum of each mouse immunized with the prophylactic vaccine or therapeutic vaccine were measured as previously described [50]. Briefly, after coating 96-well ELISA plates (Corning Inc., Corning, NY, USA) with 1 µg/ml CFA (100 µl/well) for 2 h at room temperature (RT), the plates were washed two times with PBS containing 0.05% Tween 20 (PBS-T) and blocked with 5% bovine serum albumin (BSA, 200 µl/well) for 2 h at RT. Sera from BALB/c mice were diluted from 10^−1^ to 10^−5^ with 1% BSA containing 0.05% Tween 20 (BSA-T). After three washes with PBS-T, diluted sera (100 µl/well) was added and incubated overnight at 4°C The total IgG, IgG1, IgG2a, and IgG2b (1:3000) and horseradish peroxidase (HRP, 1:250) were diluted in 1% BSA-T, respectively. Then, the plates were washed four times with PBS-T and antibodies (100 µl/well) was added to the plates and incubated for 1 h at RT. After four washes with PBS-T, diluted HRP (100 µl/well) was added to the plates and incubated for 1 h at RT. The plates were then washed six times with PBS-T. Subsequently, TMB solution (100 µl/well) was added to the plates and incubated until the color changed to blue through a reaction. Then, a 1 N H_2_SO_4_ solution (50 µl/well) was added to stop the reaction. The plates were analyzed using Agilent BioTek Gen 5 microplate reader (Santa Clara, CA, USA) at 450 nm. Following the evaluation of 10^−1^ to 10^−5^ of diluted sera for antibody responses, 10^−3^ of diluted sera were appropriated for detection (the detection limit of sera was 10^−4^ dilution). All antibodies used were obtained from BD Pharmingen (Franklin Lakes, NJ, USA), except the IgG antibody (Sigma–Aldrich).

### Quantification of cytokines

As described above, single cells harvested from the mouse lung and spleen were resuspended with RPMI 1640 containing 10% FBS. Then, lung and spleen single-cell preparations (1 × 10^6^ lung cells) were plated in 96-well cell culture plates and stimulated with CFA (0 μg/ml, 2 μg/ml, and 10 μg/ml). After *ex vivo* stimulation for 12 h at 37°C supernatants were harvested after centrifugation at 2,000 rpm for 3 min. Then, the produced cytokine (IFN-γ, TNF-α, IL-2, IL-17A, and IL-10) concentrations were measured using commercial ELISA kits (Invitrogen and BioLegend) according to the manufacturers’ protocol. Briefly, we diluted capture antibodies (1:250) in coating buffer and coated the wells of 96-well ELISA plates with coating buffer containing capture antibodies (100 µl/well) for 2 h at RT. Then, the plates were washed three times with PBS-T and blocked with assay diluent (200 µl/well) for 2 h at RT. The details of the top concentration of each standard and dilution factor of each sample were shown in Supplementary Table S3. After three washes with PBS-T, diluted standards and samples (100 µl/well) were added and incubated overnight at 4°C The HRP (1:250) and detection antibodies (1:250) were diluted in assay diluent, respectively. Then, the plates were washed four times with PBS-T and detection antibodies (100 µl/well) was added to the plates and incubated for 1 h at RT. Following four washes with PBS-T, diluted HRP (100 µl/well) was added to the plates and incubated for 1 h at RT. The plates were then washed six times with PBS-T. Subsequently, TMB solution (100 µl/well) was added to the plates and incubated until the color changed to blue through a reaction. Then, a 1 N H_2_SO_4_ solution (50 µl/well) was added to stop the reaction. The plates were analyzed using an Agilent BioTek Gen 5 microplate reader at 450 nm.

### Analysis of surface molecule expression in bone marrow-derived dendritic cells

Bone marrow-derived dendritic cells (BMDCs) from C57BL/6 were differentiated in RPMI 1640 supplemented with 10% FBS, 1% penicillin/streptomycin (P/S; Sigma-Aldrich), 50 µM β-mercaptoethanol (ThermoFisher Scientific) and 20 ng/ml GM-CSF and 5 ng/ml IL-4 (JW CreaGene Inc., Seongnam, Gyeonggi, South Korea), as described in previous study [39,51]. After 24 h treating BMDCs (1 x 10^6^ cells/well) with LPS (100 ng/ml; InvivoGen), GLA (50 ng/ml; Avanti Polar Lipids, Inc. Alabaster, AL, USA), CDG (200 ng/ml), or GLA+CDG, cells were stained with PE/Dazzle-conjugated anti-CD11c, FITC-conjugated anti-CD40, BV421-conjugated anti-CD86, PE-conjugated anti-MHC-I and APC-Cy7-conjugated anti-MHC-II for 30 min at 4°C Then cells were washed three times with PBS containing 2% FBS followed by resuspension with PBS containing 2% FBS. Expression of surface molecules was analyzed using a CytoFLEX S flow cytometer and FlowJo software, as described above. The detailed information of antibodies was summarized in Supplementary Table S2.

### Analysis of *in* *vitro* T cell polarization

Spleen cells from 6- to 8-week-old, specific pathogen-free, female C57BL/6 OT-II T-cell receptor (TCR) transgenic mice were kindly provided by Dr. Sang-Jun Ha (Yonsei University, Seoul, South Korea). Then, CD4^+^ T cells were isolated from spleen cell suspensions of OT-II mice using a MACS column (Miltenyi Biotec, Bergisch Gladbach, Germany) according to the manufacturers’ protocol. Subsequently, CD4^+^ T cells were cocultured with BMDCs (50 ng/ml GLA, 200 ng/ml CDG, 2 × 10^5^ cells/well) pulsed with OVA_323-339_ peptide (synthesized from AbFrontier; Seoul, South Korea) at a DC:T cell ratio of 1:5 for 3 days. Cocultured T cells from all groups were stained with fluorescent dye-conjugated antibodies, listed in Supplementary Table S2 and analyzed for the cell polarization using a CytoFLEX S flow cytometer and FlowJo software, as described above.

### Bacterial CFU and lung inflammation analysis

Following Mav challenge, the left lung lobe and half of the spleen harvested from mice euthanized by CO_2_ were homogenized. Afterward, serially diluted tissue lysates were spotted onto 7H10 agar containing 10% OADC and 0.5% amphotericin B (Sigma–Aldrich), and the number of CFUs was determined after three weeks of cultivation at 37°C To assess lung inflammation, the right superior lobe was fixed in 10% neutral buffered formalin and embedded in paraffin. Four- to five-micrometer sections were evaluated for the severity of lung inflammation by hematoxylin and eosin (H&E) staining and scanned using an Aperio ScanScope slide scanner (Aperio AT2; Leica Biosystems, Wetzlar, Germany). As previously described [50], all inflamed lung areas were determined through the ImageJ program (National Institutes of Health, Bethesda, MD, USA) and are represented as both mm^2^ and percentage values.

### Intracellular anti-Mav activities of recombinant in response to IFN-γ and IL-17A

After the differentiation of bone marrow-derived macrophages (BMDMs) from BALB/c mice in Dulbecco’s modified Eagle’s medium (Biowest) containing with 10% FBS, 1% P/S and 10% L929 supernatant, BMDMs were used to assess anti-Mav activities of recombinant IFN-γ and recombinant IL-17A (R&D Systems, Minneapolis, MN, USA) as previously described [39,46]. Briefly, BMDMs (4 × 10^5^ cells/ml) were cultivated in a 48-well cell culture plate for a day, infected with Mav SMC #7 at multiplicity of infection of 3 during a 4 h pre-treatment, and washed with Dulbecco’s PBS (DPBS; Biowest). The cells were cultivated with or without cytokines in triplicate wells for 3 days and lysed with 0.05% Triton X-100. Then, the serially diluted lysates with DPBS were spotted four times per well on 7H10 agar plates containing 10% OADC to quantify the number of CFUs. CFUs were determined after three weeks of cultivation at 37°C and presented as the mean CFU values along with the S.Ds per ml of BMDMs. At least two independent experiments were performed.

### Statistical analysis

The significance levels for the comparisons between groups or among groups were determined by unpaired *t test* or one-way ANOVA with a *post hoc* Tukey test, respectively, by GraphPad Prism version 7.00 (GraphPad Software, La Jolla, CA, USA, www.graphpad.com) and are represented as the mean value along with the standard deviation (S.D.). A *p* value less than .05 was regarded as indicating statistical significance.

## Results

### Comparison of immunogenicity between subunit vaccines formulated with different adjuvants

Although the protective immune correlates of the host against MAC infection have not been fully elucidated to date, two studies recently documented that ID91 and Ag85B antigen-specific multiple cytokine-expressing CD4^+^ T cells [25] or the reciprocal induction of both antigen-specific Th1 and Th17 cells are important for the control of Mav infection [39].

Thus, we attempted to identify the adjuvant combination capable of inducing Th1 and Th17 immune responses, which are known to play synergistically protective roles in the vaccine-induced control of mycobacterial infections in various models [52–55]. As the first step, we established chronic progressive Mav pulmonary infection (Mav-PI) models with two different mouse strains, C57BL/6 and BALB/c, which have been reported to have different susceptibilities to Mav infection and responses to antibiotic therapy [56,57], via the aerosol route with Mav SMC #7. The mice were euthanized to compare the bacterial loads and histopathologies at 10 weeks post-infection (Supplementary Figure S1A). As previously reported, BALB/c mice were more vulnerable to Mav SMC #7 infection than C57BL/6 mice, as they exhibited significantly higher bacterial loads (*p* < .05) in their lungs and more extensive pulmonary inflammation (*p* < .01) (Figure S1B–D). These findings led us to employ BALB/c as a murine model of severe and progressive disease for subsequent experiments. Next, we compared and investigated the immunogenicity of CFA+GLA-SE and CFA+GLA-SE/CDG.

Following the final immunization (Figure 1a) and *ex vivo* stimulation of single of homogenized lung (Figure 1b–e) or spleen (Supplementary Figure S3A–D) cells with 10 µg CFA, the CFA+GLA-SE-immunized group produced significantly more double-positive IFN-γ^+^IL-2^+^ (*p* < .01 in the lung and *p* < .001 in spleen) and IFN-γ^+^TNF-α^+^ (*p* < .01 in the lung and *p* < .001 in spleen) producing T cells than the group immunized with GLA-SE alone (referred to as the control). Notably, the level of Mav CFA-specific IFN-γ^+^IL-2^+^- and IFN-γ^+^TNF-α^+^-producing CD4^+^ T cells upon CDG addition was increased compared to that in the CFA+GLA-SE-immunized group (*p* < .0001 in both organs). In addition, the CFA+GLA-SE/CDG-immunized group elicited significantly more Mav CFA-specific IFN-γ^+^IL-17A^+^-producing CD4^+^ T cells than the control group (*p* < .0001 in both organs). In particular, the induction of these CD4^+^ T cells in the spleen was markedly enhanced in the CFA+GLA-SE/CDG-immunized group compared to that in the CFA+GLA-SE-immunized group (Figure 1b,c and Supplementary Figure S3A,B). Thus, in accordance with the FACS gating strategy (Supplementary Figure S2), higher frequencies of multifunctional Mav CFA-specific IFN-γ^+^TNF-α^+^IL-2^+^IL-17A^+^, IFN-γ^+^TNF-α^+^IL-2^+^-, IFN-γ^+^TNF-α^+^IL-17A^+^-, and IFN-γ^+^TNF-α^+^-expressing CD4^+^ T cells were detected in the lungs and spleens of the CFA+GLA-SE/CDG-immunized group than in the CFA+GLA-SE-immunized and control group (Figure 1d and Supplementary Figure S3C); moreover, the quality of Mav CFA-specific CD4^+^ T cells, including quadruple- and triple-positive cells in the lung and triple- and double-positive cells in the spleen, was increased in the CFA+GLA-SE/CDG-immunized group (Figure 1e and Supplementary Figure S3D). Collectively, we revealed a shift toward higher CD4^+^ T cell multifunctionality upon the addition of CDG.

During BCG vaccination, antibodies can activate T cells and induce cytotoxic responses [58]. Fletcher *et al*. reported that BCG vaccination with MVA85A increased Ag85A-specific IgG levels and stimulated protection against TB in South African infants who were vaccinated with BCG [59]. Hence, the CFA+GLA-SE-immunized group and CFA+GLA-SE/CDG-immunized group displayed robust CFA-specific total IgG (*p* < .001 for the CFA+GLA-SE-immunized group and *p* < .0001 for the CFA+GLA-SE/CDG-immunized group), IgG1 (*p* < .05 for the CFA+GLA-SE-immunized group and the CFA+GLA-SE/CDG-immunized group), IgG2a (*p* < .0001 for the CFA+GLA-SE-immunized group and the CFA+GLA-SE/CDG-immunized group) and IgG2b (*p* < .001 for the CFA+GLA-SE-immunized group and *p* < .0001 for the CFA+GLA-SE/CDG-immunized group) responses compared to the group immunized with GLA-SE alone (Supplementary Figure S4). Taken together, these results showed that the systemic humoral responses in terms of serum antibody levels induced after immunization with the subunit vaccines did not differ between the two vaccination groups except for the IgG2b response regardless of the adjuvant composition.

### Comparative analysis of cytokine patterns between subunit vaccines formulated with different adjuvants

**Figure 2:**
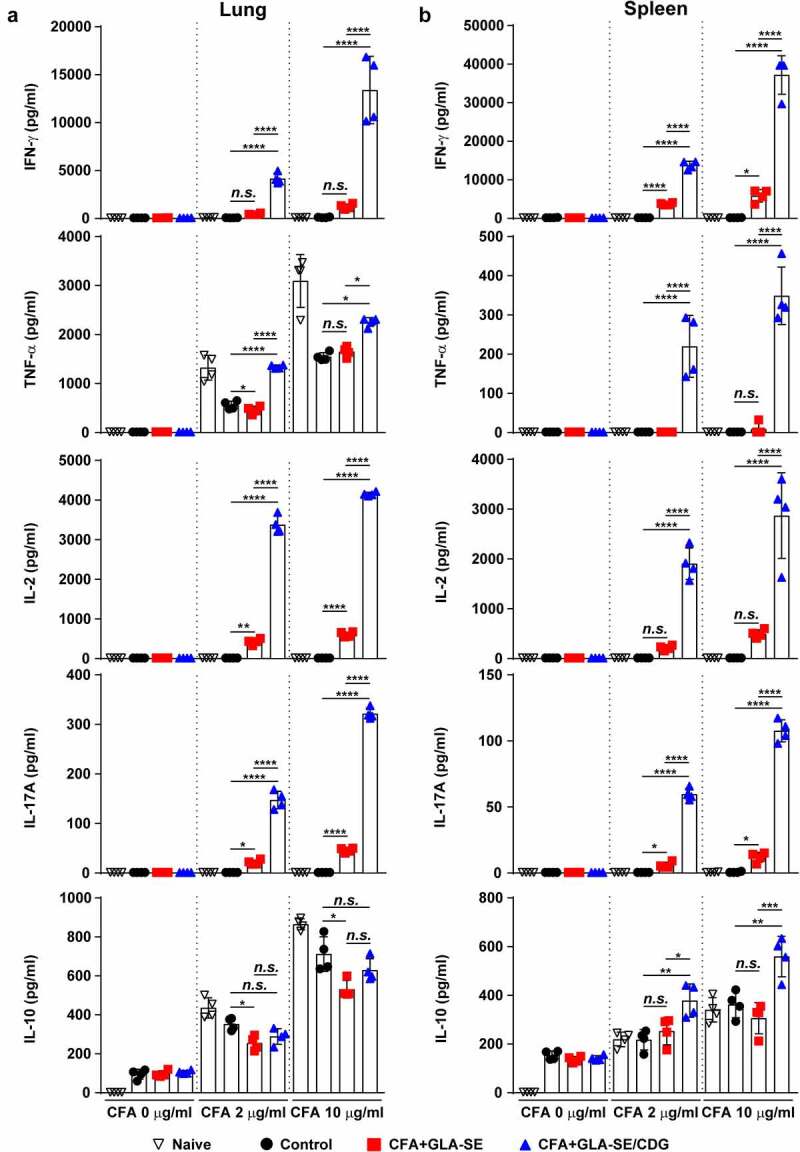
Cytokine profiles of lung and spleen single cells after *ex vivo* stimulation with CFA. At four weeks after the last immunization, mice were sacrificed, and (a) lung single cells and (b) spleen single cells harvested from each group (*n* = 4) were stimulated with CFA (0, 2, or 10 µg/ml) at 37°C for 12 h. Secreted cytokines in the collected supernatants were quantified by ELISA, and the data are represented as a scatter plot with bars. Statistically significant differences among all groups in (a) and (b) were determined by one-way ANOVA with Tukey’s multiple comparison test, and the results are presented as the means value along with the S.Ds. *p <.05, **p <.01, ***p <.001, ****p <.0001 and *n.s.*: not significant. The representative results are shown from a single *in vivo* experiment. CFA, culture filtrate antigen; Control, GLA-SE immunization alone; GLA-SE, glucopyranosyl lipid a adjuvant formulated in a stable oil-in-water emulsion; GLA-SE/CDG, GLA-SE plus cyclic-di-GMP.

Subsequently, we analyzed the production of cytokines correlated with CD4^+^ T cell phenotypes between the vaccination groups. Consistent with the enhanced multifunctionality of CD4^+^ T cells enhanced by CFA+GLA-SE/CDG immunization, the production of cytokines associated with Th1 and Th17 immunity (IFN-γ, TNF-α, IL-2, and IL-17A) in the lungs and spleens was markedly augmented upon *ex vivo* stimulation with CFA in an antigen dose-dependent manner compared with that in the CFA+GLA-SE-immunized and control groups (Figure 2a,b). While the level of the Th2 cytokine IL-10 was higher in the spleens of the CFA+GLA-SE/CDG-immunized group than in the spleens of the CFA+GLA-SE-immunized and control groups, this tendency was not observed in the lungs upon CDG addition (Figure 2). In addition, we observed that GLA+CDG *in vitro* stimulation induced enhanced maturation phenotypes in BMDCs by significantly upregulating CD40, CD86, and MHC-I except for MHC-II compared to those induced by GLA- or CDG-alone stimulation (Supplementary Figure S5A). Based on phenotypical maturation, we further investigated whether GLA+CDG-mediated BMDC contributed to T cell polarization. Interestingly, OVA_323-330_-pulsed GLA+CDG-stimulated BMDCs possessed an increased capacity of directing T cells toward Th1/Th17 phenotype as evidenced by up-regulated RORγt and T-bet expression (Th1- and Th17-associated transcription factors) compared to those induced by either OVA_323-330_-pulsed GLA- or CDG-stimulated BMDCs (Supplementary Figure S5B). Collectively, these results suggested that immunization with Mav CFA in combination with the adjuvant GLA-SE/CDG elicited more Th1/Th17 cytokine production and antigen-specific Th1/Th17 CD4^+^ T cells in the lungs than immunization with the GLA-SE adjuvant alone.

### Assessment of protection efficacy and its correlation with the maintenance of Mav CFA-specific Th1/Th17 responses

**Figure 3:**
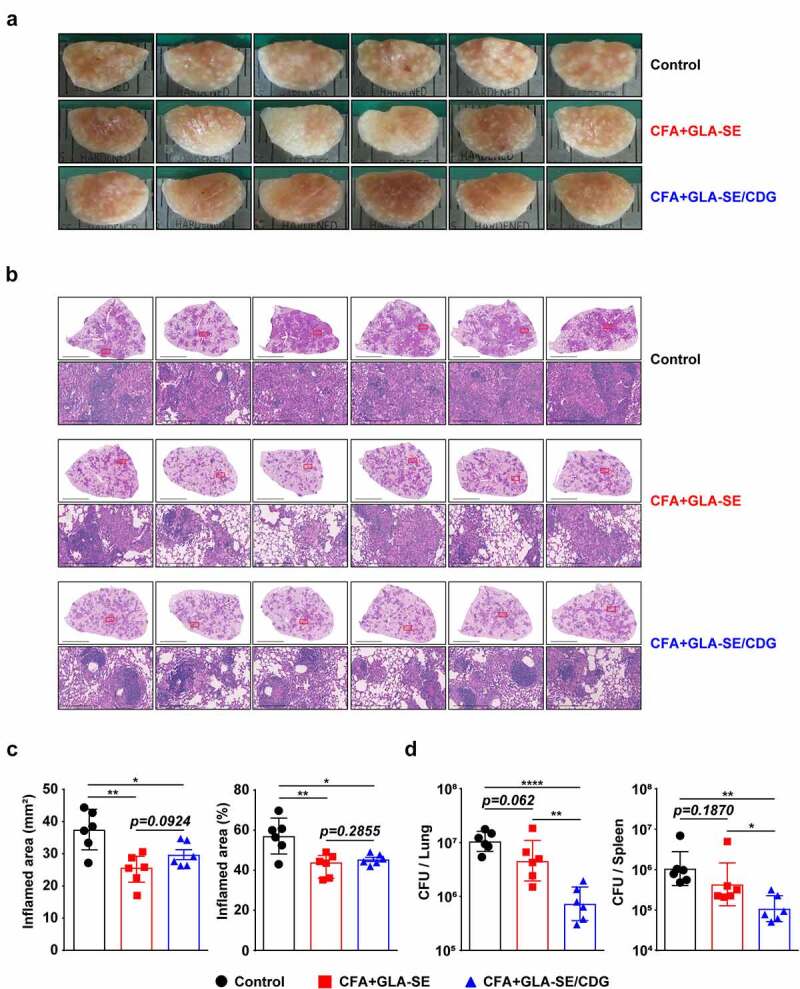
Long-Term prophylactic protective efficacies of CFA+GLA-SE and CFA+GLA-SE/CDG immunization in a murine model of chronic progressive Mav-PI at 10 weeks post-infection. At 10 weeks post-infection, after euthanization of each group of immunized and infected mice (*n* = 6), (a) gross images and (b) 10× and 200× magnification photomicrographs with H&E staining (scale bars = 3 mm and 200 µm) of the right superior lobe of infected lung tissues were obtained; those of the entire groups are displayed. Each immunized group is indicated on the right side of the images in (a) and (b). (c) Quantitative analysis of the inflamed areas in the H&E-stained lung tissues. the sizes and percentages of the lesions in (b) are presented as a scatter plot with bars. The bacterial burdens (d) in the left lung lobe and half of the spleen in the mice from each group were assessed by counting viable bacterial colonies grown on 7H10-OADC agar plates, and the data are shown as in scatter plot with bars. Statistically significant differences among all groups in (c) and (d) were calculated by the unpaired *t test*, and the results are represented as the mean values along with the S.Ds. *p <.05, **p <.01 and ****p <.0001. The representative results are shown from a single *in vivo* experiment. CFA, culture filtrate antigen; Control, GLA-SE immunization alone; GLA-SE, glucopyranosyl lipid a adjuvant formulated in a stable oil-in-water emulsion; GLA-SE/CDG, GLA-SE plus cyclic-di-GMP.

Ultimately, the protective efficacies of CFA+GLA-SE- and CFA+GLA-SE/CDG-immunization were compared based on bacterial burdens and lung inflammation, and the protective correlates were investigated. Compared with the control group (GLA-SE adjuvant alone and Mav-infected group), both the CFA+GLA-SE- (*p* < .01) and CFA+GLA-SE/CDG-immunized groups (*p* < .05) displayed reduced granuloma-like lesion formation and lung inflammation at 10 weeks post-infection; however, significant differences were not exhibited between the two groups (Figure 3a–c). Interestingly, the CFA+GLA-SE/CDG-immunized group exhibited significantly reduced viable Mav numbers in the lungs (*p* < .0001), while CFA+GLA-SE immunization was not effective at controlling bacteria (Figure 3d). Although CFA+GLA-SE/CDG immunization conferred enhanced protection in the spleen compared to that with the control (*p* < .01), the bacterial loads in the CFA+GLA-SE/CDG-immunized group were not significantly reduced compared to those in the CFA+GLA-SE-immunized group (Figure 3d). Taken together, immunization with CFA+GLA-SE/CDG resulted in improved protection in terms of the reduced bacterial load at 10 weeks after Mav SMC #7 infection.

**Figure 4:**
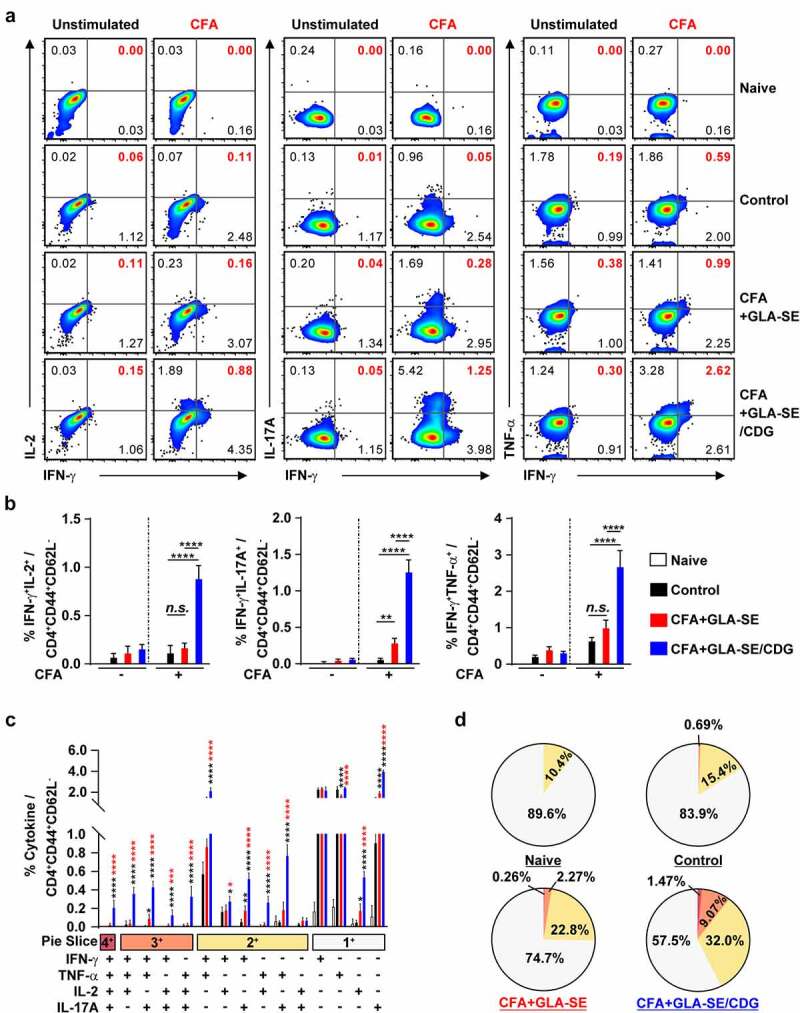
Quantitative and qualitative analyses of the long-lasting induction of Mav CFA-specific multifunctional CD4^+^ T cells after CFA+GLA-SE or CFA+GLA-SE/CDG immunization in a murine model of chronic progressive Mav-PI at 10 weeks post-infection. At 10 weeks post-infection, the mice were sacrificed, and lung cells harvested from each immunized group (*n* = 6) and naïve group (*n* = 4) were stimulated by GolgiPlug and GolgiStop with or without 10 µg/ml CFA at 37°C for 9 h. The frequencies of Mav CFA-specific IFN-γ^+^IL-2^+^, IFN-γ^+^IL-17A^+^ or IFN-γ^+^TNF-α^+^-expressing CD4^+^CD44^+^CD62 L^−^ T cells were assessed after staining of intracellular cytokines and are presented as (a) pseudocolor dot plots and (b) summary bar graphs. (c) The percentages of total CD4^+^CD44^+^CD62 L^−^ T cells with differential production of IFN-γ, IL-2, IL-17A and TNF-α in response to CFA stimulation among lung single cells were determined among groups and are presented as bar graphs. (d) The values of the proportions of quadruple- (4+, crimson), triple- (3+, orange), double- (2+, yellow), and single-function (1+, light gray) CD4^+^CD44^+^CD62 L^−^ T cells expressing IFN-γ, IL-2, IL-17A and TNF-α in each immunized group and naïve group are illustrated as pie charts. Statistically significant differences among all groups in (b) and (c) were determined by one-way ANOVA with Tukey’s multiple comparison test, and the results are presented as the mean values along with the S.Ds. *p <.05, **p <.01, ****p <.0001 and *n.s.*: not significant. The asterisks in (c) represent significant differences between groups: black, control group vs. CFA+GLA-SE/CDG group; red, CFA+GLA-SE group vs. CFA+GLA-SE/CDG group. The representative results are shown from a single *in vivo* experiment. CFA, culture filtrate antigen; Control, GLA-SE immunization alone; GLA-SE, glucopyranosyl lipid a adjuvant formulated in a stable oil-in-water emulsion; GLA-SE/CDG, GLA-SE plus cyclic-di-GMP.

**Figure 5:**
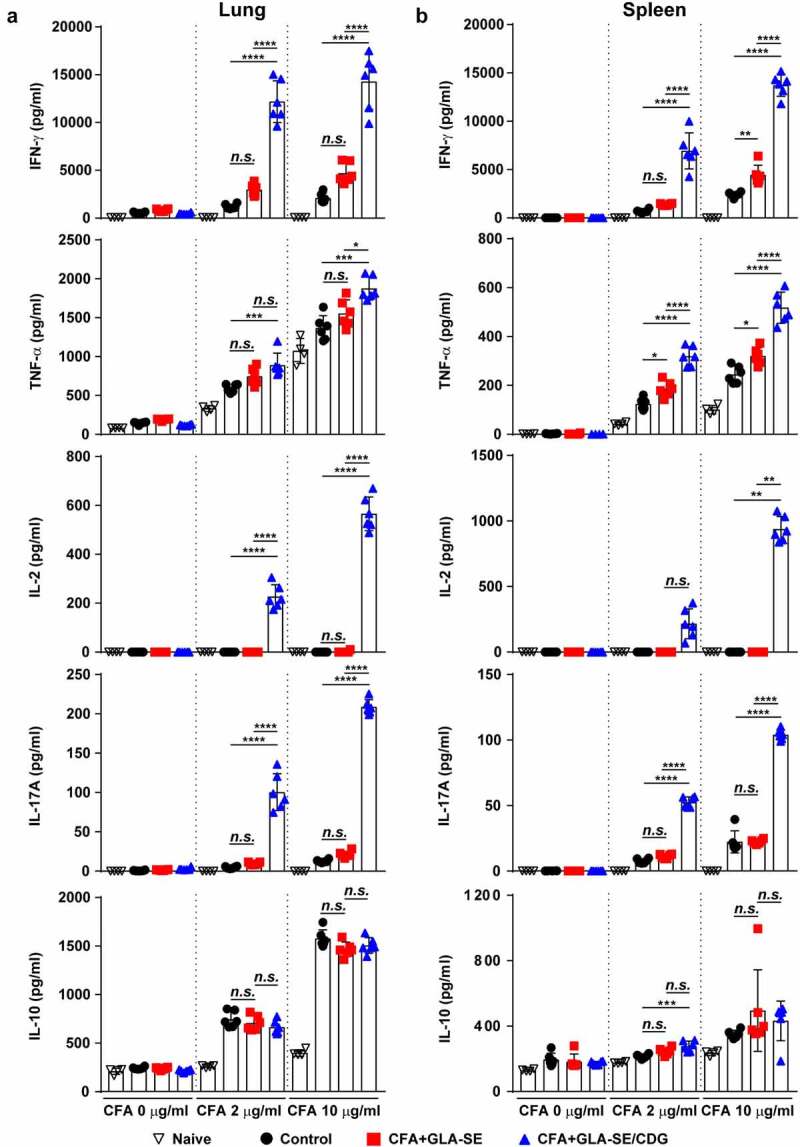
Cytokine profiles of lung and spleen single cells of mice with chronically progressive Mav-PI after *ex vivo* stimulation with CFA at 10 weeks post-infection. At 10 weeks post-infection, mice were sacrificed, and (a) lung single cells and (b) spleen single cells harvested from each immunized group (*n* = 6) and naïve group (*n* = 4) were stimulated with CFA (0, 2, or 10 µg/ml) at 37°C for 12 h. Secreted cytokines in the collected supernatants were quantified by ELISA, and the data are represented as a scatter plot with bars. Statistically significant differences among all groups in (a) and (b) were determined by one-way ANOVA with Tukey’s multiple comparison test, and the results are presented as the mean values along with the S.Ds. *p <.05, **p <.01, ***p <.001, ****p <.0001 and *n.s.*: not significant. The representative results are shown from a single *in vivo* experiment. CFA, culture filtrate antigen; control, GLA-SE immunization alone; GLA-SE, glucopyranosyl lipid a adjuvant formulated in a stable oil-in-water emulsion; GLA-SE/CDG, GLA-SE plus cyclic-di-GMP.

Hence, to determine whether the maintenance of Mav CFA-specific Th1/Th17 responses within the context of CFA+GLA-SE/CDG immunization is a relevant strategy for controlling Mav pathogenesis, we assessed immune responses in the lungs and spleens of mice induced by Mav SMC #7 infection for 10 weeks. After *ex vivo* Mav CFA stimulation of single cells from the lungs or spleens of Mav SMC #7-infected mice in the CFA+GLA-SE-immunized group, double-positive CD4^+^ T cells expressing IFN-γ^+^IL-17A^+^ (*p* < .01 in the lung) were remarkably enriched compared with those in the control group mice. Remarkably, long-lasting induction of Mav CFA-specific IFN-γ^+^IL-2^+^-, IFN-γ^+^IL-17A^+^-, and IFN-γ^+^TNF-α^+^-expressing CD4^+^ T cells upon CDG addition (*p* < .0001) in the lung and Mav CFA-specific IFN-γ^+^IL-2^+^- and IFN-γ^+^TNF-α^+^-expressing CD4^+^ T cells upon CDG addition (*p* < .0001 and *p* < .01) in the spleen were observed compared with those in the CFA+GLA-SE-immunized and control groups (Figure 4a,b and Supplementary Figure S6A,B). Importantly, the frequencies of multifunctional CFA-specific T cells producing IFN-γ^+^TNF-α^+^IL-2^+^IL-17A^+^, IFN-γ^+^TNF-α^+^IL-2^+^, IFN-γ^+^TNF-α^+^IL-17A^+^, IFN-γ^+^IL-2^+^IL-17A^+^, IFN-γ^+^TNF-α^+^, IFN-γ^+^IL-2^+^, and IFN-γ^+^IL-17A^+^ in the lungs and those of IFN-γ^+^TNF-α^+^IL-2^+^ producing T cells in the spleens of the CFA+GLA-SE/CDG-immunized group were highly detected compared to those in the CFA+GLA-SE-immunized and control groups (Figure 4c,d and Supplementary Figure S6C,D), suggesting that the CFA+GLA-SE/CDG immunization-derived multifunctionality of CD4^+^ T cells was durably sustained at 10 weeks post-infection. Next, we further investigated long-lasting humoral responses for CFA-specific total IgG (*p* < .0001 for CFA+GLA-SE-immunized group and *p* < .001 for CFA+GLA-SE/CDG-immunized group), IgG1 (*p* < .001 for CFA+GLA-SE-immunized group and *p* < .01 for CFA+GLA-SE/CDG-immunized group), IgG2a (*p* < .0001 for both immunization groups) and IgG2b (*p* < .001 for CFA+GLA-SE-immunized group and *p* < .0001 for CFA+GLA-SE/CDG-immunized group) by comparing with those of the control groups, but no enhancement upon CDG addition was observed prior to infection (Supplementary Figure S7), indicating that systemic humoral responses in terms of serum antibody levels might have a minimal role in preventative protection against Mav-PI. Moreover, the profiles of cytokine production after *ex vivo* Mav CFA stimulation of single lung and spleen cells were used to determine whether Th1/Th17 responses were maintained (Figure 5). The patterns of IFN-γ, TNF-α, IL-2, and IL-17A secretion, but not IL-10, were remarkably increased upon CFA stimulation in a dose-dependent manner compared with those in the CFA+GLA-SE-immunized and control groups (Figure 5). For further assessment of the involvement of Th1/Th17 responses in protecting against Mav infection, we conducted *in vitro* experiment to evaluate the role of IL-17A and IFN-γ in terms of controlling intracellular Mav growth (Supplementary Figure S8). Interestingly, treatment with 20 ng/ml IL-17A alone did not show any efficacy, while treatment with 20 ng/ml IFN-γ alone exhibited anti-Mav activity in Mav-infected BMDMs. In addition, treatment with the combination of IFN-γ and IL-17A at 10 ng/ml each (a half dose of the single treatment) and the combination of IFN-γ and IL-17A at 20 ng/ml each remarkably inhibited intracellular Mav growth compared with 20 ng/ml IFN-γ alone. Based on these results, IL-17A contributed to enhanced control of intracellular growth of Mav in cooperation with IFN-γ. Taken together, these analyses indicated that the magnitude and sustainability of Mav CFA-specific Th1/Th17 responses could protect against Mav-PI and disease progression.

### Advanced application of Th1/Th17-inducing subunit immunization as an adjunctive therapeutic measure during antibiotic treatment

**Figure 6:**
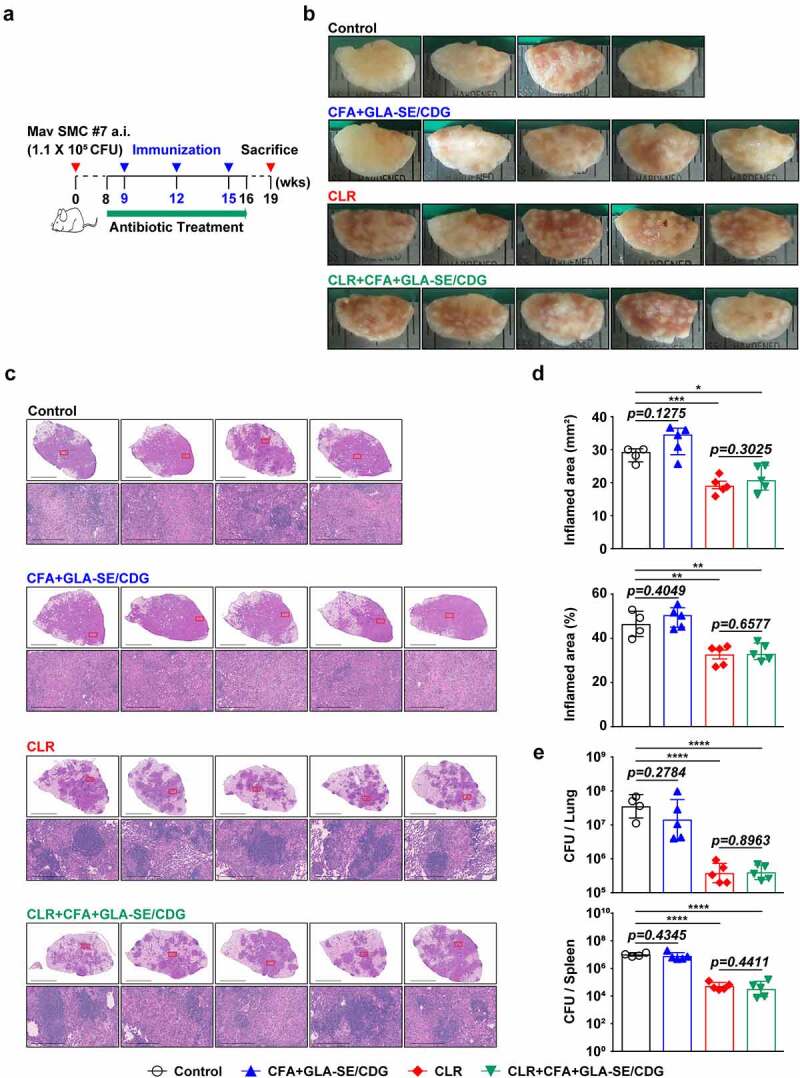
Comparative assessment of the protective efficacies of antibiotic treatment and therapeutic vaccination in a murine model of chronic progressive Mav-PI at 19 weeks post-infection. (a) Scheme of the *in vivo* experiment. At eight weeks after BALB/c infection with Mav SMC #7, eight weeks of daily therapy was initiated with 100 mg/kg (oral) CLR, along with immunization with CFA+GLA-SE/CDG three times at 3-week intervals. Three weeks after discontinuation of the medication, five mice in each treatment group (*n* = 5) and four mice in the infection control group were sacrificed, and their lungs were homogenized for bacterial CFU, lung inflammation and immunological assays at 19 weeks post-infection. (b) Gross images and (c) 10× and 200× magnification photomicrographs with H&E staining (scale bar = 3 mm and 200 µm) of the right superior lobe of infected lung tissues of all groups are displayed. Each treatment group is indicated in the upper part of the representative lung pathology image in (b) and (c). (d) Quantitative analysis of the inflamed areas in the H&E-stained lung tissues. The sizes and percentages of the lesions in (c) and the data are presented as scatter plots with bars. The bacterial burdens (e) in the left lung lobe and half of the spleen in each group were assessed by counting viable bacterial colonies grown on 7H10-OADC agar plates, and the data are presented as a scatter plot with bars. Statistically significant differences among all groups in (d) and (e) were calculated by the unpaired *t test*, and the results are represented as the mean values along with the S.Ds. *p <.05, **p <.01, ***p <.001, and ****p <.0001. The representative results are shown from a single *in vivo* experiment. CLR, clarithromycin; Control, untreated infection control; CFA, culture filtrate antigen; GLA-SE/CDG, glucopyranosyl lipid a adjuvant formulated in a stable oil-in-water emulsion plus cyclic-di-GMP.

Given that CFA+GLA-SE/CDG immunization conferred durable protection accompanied by enhanced Th1/Th17 immunity in the prophylactic setting, we further aimed to determine whether increasing Th1/Th17 responses could enhance the efficacy of antibiotics to better control Mav infection in a chronic progressive stage. Therefore, we examined the effect of antibiotics on the CD4^+^ T cell responses to Mav-derived CFA of infected mice and demonstrated that a therapeutic vaccination strategy using CFA+GLA-SE/CDG enhanced the responsiveness of CD4^+^ T cell reactivity during antibiotic treatment. Using the established BALB/c model of Mav SMC #7-infection, the mice were vaccinated with CFA+GLA-SE/CDG three times while orally receiving CLR administration for 8 weeks starting from 8 weeks post-infection, and analyses of the bacterial loads, histopathology and immune responses were performed at 19 weeks post-infection (Figure 6a). Interestingly, unlike in the prophylactic setting, CFA+GLA-SE/CDG immunization alone as a therapeutic strategy without CLR treatment did not ameliorate lung inflammation or induce necrotic lesion development at 19 weeks post-infection (Figure 6b–d). Moreover, no synergy in terms of reduced lung inflammation was observed in mice receiving CFA+GLA-SE/CDG therapeutic immunization during CLR treatment (referred to as CLR+CFA+GLA-SE/CDG) compared to that in mice receiving CLR monotherapy (Figure 6b–d). Furthermore, bacterial growth was significantly inhibited in the lungs and spleens in both the CLR monotherapy and CLR+CFA+GLA-SE/CDG groups, but therapeutic immunization with CFA+GLA-SE/CDG did not further reduce the bacterial loads (Figure 6e). Overall, therapeutic immunization with CFA+GLA-SE/CDG displayed no protective effects and did not function synergistically with CLR in the chronic progressive Mav-PI model.

**Figure 7:**
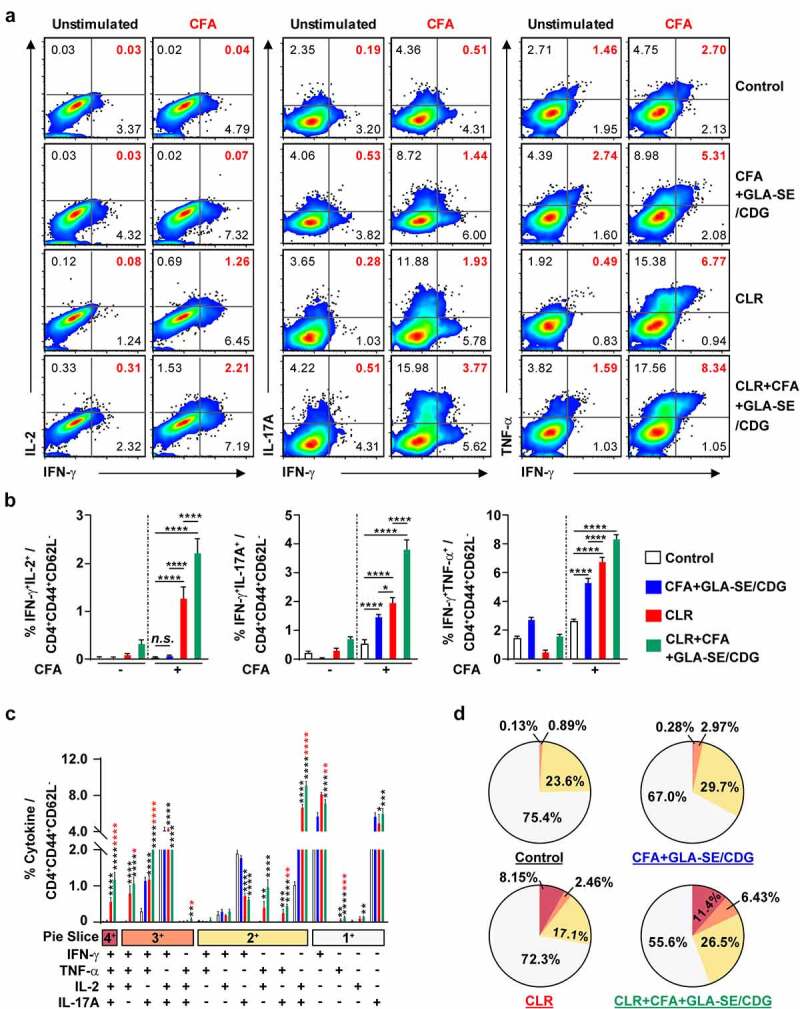
Qualitative and quantitative analyses of Mav CFA-specific multifunctional lung CD4^+^ T cells after treatment with CLR to boost CFA+GLA-SE/CDG immunization in a murine model of chronic progressive Mav-PI at 19 weeks post-infection. At 19 weeks post-infection, lung cells from sacrificed mice of each treatment group (*n* = 5) and infection control group (*n* = 4) were stimulated by GolgiPlug and GolgiStop with or without 10 µg/ml CFA at 37°C for 9 h. The frequencies of Mav CFA-specific IFN-γ^+^IL-2^+^-, IFN-γ^+^IL-17A^+^- and IFN-γ^+^TNF-α^+^-expressing CD4^+^CD44^+^CD62 L^−^ T cells were assessed after staining of intracellular cytokines and are presented as (a) pseudocolor dot plots and (b) summary bar graphs. (c) The percentages of total CD4^+^CD44^+^CD62 L^−^ T cells with differential expression of IFN-γ, IL-2, IL-17A and TNF-α in response to CFA stimulation among lung single cells were determined among groups and are presented as bar graphs. (d) The values of the proportions of quadruple- (4+, crimson), triple- (3+, orange), double- (2+, yellow), and single-function (1+, light gray) CD4^+^CD44^+^CD62 L^−^ T cells expressing IFN-γ, IL-2, IL-17A and TNF-α in each infected, immunized, and treated group are illustrated as pie charts. Statistically significant differences among all groups in (b) and (c) were determined by one-way ANOVA with Tukey’s multiple comparison test, and the results are presented as the mean values along with the S.Ds. *p <.05, **p <.01, ***p <.001, ****p <.0001 and *n.s.*: not significant. The asterisks in (c) represent significant differences between groups: black, control group vs. CLR group or CLR+CFA+GLA-SE/CDG group; red, CLR group vs. CLR+CFA+GLA-SE/CDG group. The representative results are shown from a single *in vivo* experiment. CFA, culture filtrate antigen; Control, untreated infection control; GLA-SE/CDG, glucopyranosyl lipid a adjuvant formulated in a stable oil-in-water emulsion plus cyclic-di-GMP; CLR, clarithromycin.

**Figure 8:**
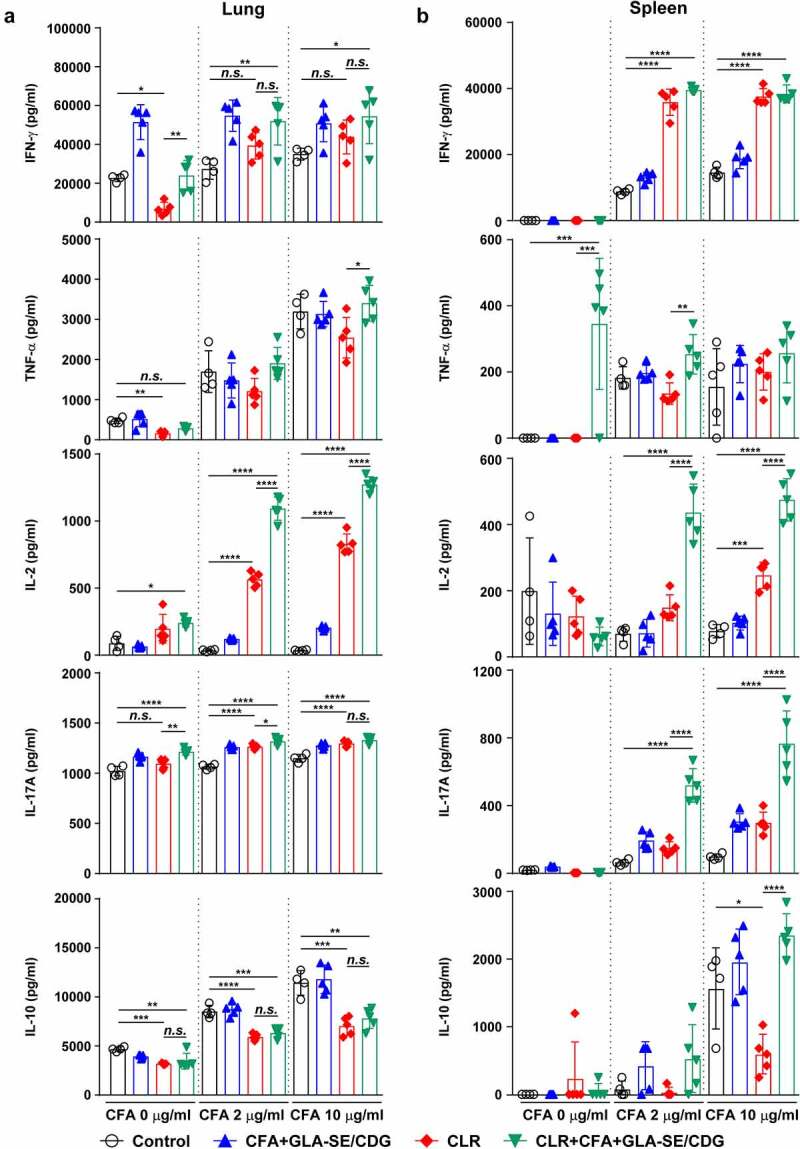
Cytokine profiles of lung and spleen single cells of mice treated with antibiotics and vaccinated with CFA+GLA-SE/CDG in a murine model of chronic progressive Mav-PI, after *ex vivo* stimulation with CFA at 19 weeks post-infection. At 19 weeks post-infection, mice were sacrificed, and (a) lung single cells and (b) spleen single cells harvested from each treatment group (*n* = 5) and infection control group (*n* = 4) were stimulated with CFA (0, 2, or 10 µg/ml) at 37°C for 12 h. Secreted cytokines in the collected supernatants were quantified by ELISA, and the results are presented as a scatter plot with bars. Statistically significant differences among all groups in (a) and (b) were determined by one-way ANOVA with Tukey’s multiple comparison test, and the results are presented as the mean values along with the S.Ds. *p <.05, **p <.01, ***p <.001, ****p <.0001 and *n.s.*: not significant. The representative results are shown from a single *in vivo* experiment. CFA, culture filtrate antigen; Control, untreated infection control; GLA-SE/CDG, glucopyranosyl lipid a adjuvant formulated in a stable oil-in-water emulsion plus cyclic-di-GMP; CLR, clarithromycin.

Therapeutic immunization with CFA+GLA-SE/CDG was employed because enhanced Th1 and Th17 immunity accompanied improved protection in the prophylactic setting, and we thus next confirmed whether Th1/Th17 responses were sustained during antibiotic treatment. The same analytical approaches as those used in the prophylactic setting were utilized at 19 weeks post-infection (Figures 7 and 8 and Supplementary Figures S9 and S10). Regardless of CLR treatment, CFA+GLA-SE/CDG immunization significantly elicited Mav CFA-specific CD4^+^CD44^+^CD62 L^−^ T cells producing IFN-γ^+^IL-17A^+^, and IFN-γ^+^TNF-α^+^ but not IFN-γ^+^IL-2^+^ in the lungs in the CFA+GLA-SE/CDG-immunized group compared with those in the infection control group (Figure 7a,b). Surprisingly, the frequencies of Mav CFA-specific IFN-γ^+^IL-2^+^-, IFN-γ^+^IL-17A^+^-, and IFN-γ^+^TNF-α^+^-producing CD4^+^ T cells were increased in the lungs in the CLR monotherapy group and were further elevated by therapeutic immunization with CFA+GLA-SE/CDG in response to *ex vivo* stimulation with CFA (Figure 7a,b). Although CLR monotherapy-derived Mav CFA-specific IFN-γ^+^IL-2^+^-, IFN-γ^+^IL-17A^+^-, and IFN-γ^+^TNF-α^+^-producing CD4^+^ T cells were not observed in the spleen, the CFA+GLA-SE/CDG immunization group, regardless of CLR treatment, exhibited significantly enhanced frequencies of Mav CFA-specific CD4^+^ T cells (Supplementary Figure S9A, B). In line with these findings, CFA-specific multifunctional IFN-γ^+^TNF-α^+^IL-2^+^IL-17A^+^-, IFN-γ^+^TNF-α^+^IL-2^+^-, and IFN-γ^+^TNF-α^+^IL-17A^+^-producing CD4^+^ T cells in both the lungs and spleens were enriched more significantly in the CLR+CFA+GLA-SE/CDG group than in the CLR monotherapy group (Figure 7c and Supplementary Figure S9C); moreover, the quality of CFA-specific T cells, including quadruple-, triple- and double-positive cells in the lung and quadruple- and triple-positive cells in the spleen, was enhanced in the CLR+CFA+GLA-SE/CDG group (Figure 7d and Supplementary Figure S9D). Upon the assessment of humoral responses, the CLR+CFA+GLA-SE/CDG group exhibited more enhanced CFA-specific IgG2a and IgG2b responses, which are generally related to Th1 responses [60], than the CLR monotherapy group (*p* < .05) (Supplementary Figure S10), but these responses did not contribute to enhancing the therapeutic efficacy against Mav infection. Furthermore, we assessed the cytokine levels in the lungs and spleens after *ex vivo* Mav CFA stimulation to demonstrate whether Th1/Th17-mediated responses were regulated (Figure 8). The frequencies of the multifunctionality of CD4^+^ T cells, including those producing Th1- and Th17-associated cytokines such as IFN-γ, IL-2, and IL-17A but not TNF-α, were increased, but those of cells expressing IL-10 were decreased in the lung and spleens in the CLR+CFA+GLA-SE/CDG group with *ex vivo* Mav CFA stimulation, compared to those in the infection control group. Moreover, IL-2 levels were increased to a greater extent in the CLR+CFA+GLA-SE/CDG group than in the CLR monotherapy group (Figure 8). Nevertheless, strategies for enhancing Th1/Th17 responses with therapeutic immunization failed to improve bacterial clearance during antibiotic treatment. As shown in Supplementary Figure S11, Th1/Th17-related cytokine-producing T cells (*p* < .001 and *p* < .0001) involved in preventative immunization were correlated with the bacterial reduction in response to CFA restimulation while no prominent correlation between Th1/Th17-related cytokine-producing T cells and bacterial burden was observed in the therapeutic vaccine challenge, suggesting that boosting T cell immunity may not be a beneficial approach, at least in a chronic Mav-PI murine model with antibiotic treatment.

## Discussion

In our study, the immunogenicity and efficacies of preventative and therapeutic immunizations were investigated using CFA from Mav clinical strain SMC #7 and different adjuvant formulations in progressive MAC-PI murine models (Supplementary Table S4). Because BALB/c mice exhibited a more severe form of disease than C57BL/6 mice according to the bacterial loads and lung inflammation, we selected the BALB/c strain for subsequent evaluation. We used CFA from Mav SMC #7 as a vaccine antigen since no definitive vaccine antigen targets have been identified. In line with Mtb studies, Mav CFA has been scrutinized as a candidate for a heterologous mixed protein vaccine (secreted antigen as a major T cell antigen target [61–63]). Robust induction of CFA-specific multifunctional CD4^+^ T cells coproducing Th1/Th17 cytokines was achieved with CFA-adjuvanted GLA-SE/CDG before and 10 weeks after Mav aerosol challenge. This immunization resulted in remarkable protection in both the lungs and spleens compared with that provided to the groups receiving GLA-SE alone and CFA+GLA-SE. However, this vaccine strategy had a less adjunctive effect on reducing the bacterial loads and pulmonary inflammation in mice receiving antibiotic treatment despite boosting CFA-specific Th1/Th17 responses to a greater extent, indicating that differential immune responses may be required according to the disease stages and vaccination goals.

Since CD4^+^ T cells are critical for protecting against Mtb infection, the enrichment of Th1 cells producing multiple proinflammatory cytokines (e.g., IFN-γ, TNF-α, and IL-2) has been the main focus in the TB vaccine field [64–67]. Considering that patients who exhibit impaired immune responses related to the IL-12/IFN-γ axis or generate autoantibodies against IFN-γ frequently progress to being afflicted by severe disseminated and extrapulmonary forms of MAC disease, Th1-type immunity is regarded as a protective determinant of MAC disease responses [68–70]. Along with the importance of Th1 responses controlling mycobacterial infection, Choi *et al*. recently described that the early induction of IL-17 production enhanced the ability of macrophages to control Mtb infection by collaborating with IFN-γ more effectively than the mere induction of IFN-γ [39]. In addition, Bak *et* *al*. recently identified that Mav-specific Th17 responses have the potential to protect against NTM-PD when accompanied by allergic asthma [34]. Furthermore, decreased influx of CD4^+^ T cells producing Th1 and Th17 responses was followed by a plateau in the bacterial burden in Mav-infected C3HeB/FeJ mice [71], indicating that Th1/Th17 responses were essential for preventing progression from an early infection phase to chronic stages of MAC infection. Similar to previous studies, the antigen-specific Th1/Th17 responses generated herein were enhanced by CFA+GLA-SE/CDG immunization and maintained at 10 weeks post-infection, resulting in decreases in the bacterial loads and lung inflammation, and consequently slowed the progression of MAC-PI. Since the diagnosis and treatment of MAC-PD are based on positive acid-fast bacilli smear of sputum samples and isolation and identification of the microorganism in cultures [22,72–74], a lower bacterial burden is correlated with better clinical outcome in patients with MAC-PD. However, it should be noted that the degree of lung inflammation is not plainly associated with the efficacy of immunization. Our observations are consistent with previous reports [25,34,75] in which lung pathology scores did not correlate with the bacterial loads in the context of Mav infection, suggesting that protective immune responses minimally require some immune cell components and antigen-specific but vigorous immune responses.

One interesting finding in our present study is that the enhancement of reciprocal Mav CFA-specific Th1/Th17 CD4^+^ T cell responses substantially contributed to the prevention of disease progression after Mav SMC #7 infection, but these cells had an almost negligible adjunctive therapeutic effect when applied in combination with antibiotic treatment. Indeed, macrolides in other diseases or in the context of inflammatory conditions or agents (chronic rhinosinusitis, COVID-19, and LPS) suppress proinflammatory responses [76–79]; however, changes in immune responses induced by macrolide treatment for MAC-PD have not been reported. Due to the limitations of the associated data, we first showed that mice in the CLR-treated groups spontaneously induced more vigorous antigen-specific Th1/Th17 responses than those in the untreated group. However, additional immunization during antibiotic treatment had no beneficial effect on reducing the bacterial loads or lung inflammation and resulted in even more inflammatory lung lesions than the chemotherapy alone despite inducing significantly more Mav CFA-specific multifunctional CD4^+^ T cells coproducing Th1/Th17 cytokines. These data indicated that differential approaches may be required according to the disease progression stage, which is consistent with previous research showing that immunization-induced cellular immunity enhances the pathological condition or triggers the reactivation of persistent bacteria in the lung [80]. More recently, Costa *et al*. demonstrated that enhanced antibiotic clearance of Mtb was not achieved via either the adoptive transfer of ESAT-6- or Ag85B-specific Th1-polarized CD4^+^ T cells (C7 and P25 cells, respectively) or the intravenous injection of the ESAT-6_1-20_ or Ag85B_280-294_ peptide during the initial month of chemotherapy for the purpose of boosting Th1 responses [81]. Given that similar results were observed in our study, approaches to boosting Th1/Th17 responses during the initial phase of antibiotic treatment against antigens that are highly expressed in actively dividing bacteria can feasibly be reconsidered. This may be attributed to the fact that the antibiotic-derived elimination of replicating bacteria might precede the targeting of Th1/Th17 responses [81]. In fact, paradoxical responses (PRs) can occasionally develop during mycobacterial infection, leading to acute inflammation and increased bacterial burden and thus to a need for further surgical treatment [82–86]. However, little is known about the occurrence of PRs at the time of MAC-PD treatment. Recently, Huh *et* *al.* reported that PRs occurred during the antibiotic treatment course in patients with MAC-PD at an early stage, particularly within 6 months after the initiation of treatment [87]. MAC-associated immune reconstitution inflammatory syndrome (IRIS) can also appear as “unmasking” IRIS in HIV patients with asymptomatic and undiagnosed MAC infection or as “paradoxical” IRIS in people with previously established MAC disease [88,89]. Taken together, these results suggest that more precise vaccination regimens with therapeutic immunization should be further designed with a greater understanding of necessary immune responses to complement chemotherapy as an adjunctive approach to the treatment of MAC-PD.

Our current study has several limitations. Our current study has several limitations. First, because of the possibility of nonspecific CFA effects on vaccine efficacy, further identification of the major constituents of the Mav CFA is necessarily required. Although we determined the CFA-specific Th1/Th17 responses, we were unable to indicate which protein in CFA was the immunodominant T cell antigen contributing to protection against Mav infection and which unspecific protein might be related to failed therapeutic vaccination. As we expected, the Ag85 protein might be one of the major constituents since the Ag85 protein (Ag85A, B or C) is well-defined protein in the *Mycobacterium* genus and recombinant fusion protein ID91 containing Ag85B recently exhibited effectiveness in protecting against Mav infection [25,63,90]. Therefore, the identification of the major constituents of the CFA will help us to dissect immunologically protective correlates more specifically and optimize the regimen in a future study. Second, evaluation with adjuvants other than GLA-SE and CDG is required to evaluate the general implementation and conclusion of our findings on the effect of adjuvants in combination with the induction of Th1/Th17 effector/memory T cell production, ultimately leading to the improvement of protective immunity in other preclinical models, including outbred mice and nonhuman primates. Third, the timing of therapeutic immunization is crucial to elicit optimal protective immune responses during antibiotic treatment. Notably, our current therapeutic approaches failed to exert a meaningful effect on late, progressive stages of the disease. As discussed above, this failure may be attributed to the fact that innate immunity at this stage with relatively maximal bacterial loads might affect vaccine responses [91]. In this context, therapeutic immunity may be applied after drug treatment is complete to elicit the most effective immune effector responses because immune function may be reinvigorated after periods of overt disease and suppression associated with bacterial replication [92]. Although bacterial CFUs and lung inflammation are the most prominent features of a mouse model of progressive Mav-PI, our therapeutic immunization did not confer additional protective effects, suggesting that the contribution of augmented Th1/Th17 cells to advanced chronic disease outcomes should be further investigated. Forth, although we demonstrated a role for Th1/Th17 cells induced by GLA-SE/CDG in vaccine-induced protective immunity against Mav-PI in the preventative setting, the precise mechanisms of Th17 responses in the generation of optimal anti-Mav immunity are still unknown. Finally, Th17 cells are identified by unique plasticity and heterogeneity and exert both pathogenic and preventative effects against Mav infection [93,94]. Although we defined Th17 responses using identical markers in preventative and therapeutic immunization, Th17 cells may have heterogeneous plasticity and play different roles in early protection or chronic lung inflammation. Additionally, the biological activities of IL-17 differ depending on its binding to its multimeric receptors [95–97]. Thus, whether the Th17 phenotype and the relevant functions are identical in the context of the disease stage should be investigated in future studies. Nevertheless, the results of this study provide fundamental insights into vaccine-induced defensive T cell-mediated immunity against Mav-PI. In addition, we documented how adjuvant formulations in combination induce differential effector CD4^+^ T cell immune responses in mice. Furthermore, by eliciting T cell-polarizing cytokine production (e.g., IFN-γ, IL-2, and IL-17A), GLA-SE/CDG immunization in combination differentially programs T cell subsets in the lungs. Finally, the combination of adjuvants allows the selective utilization of the most desirable properties while mitigating the less desirable effects of individual adjuvants.

Taken together, our data demonstrate that vaccine-induced immune responses differ according to the purpose of prophylactics and immunotherapeutic adjuncts used in combination with antibiotics. Enrichment of CFA-specific multifunctional CD4^+^ T cells coproducing Th1/Th17 cytokines represents a promising approach for preventing Mav infection and slowing disease progression, but these increased responses also exerted no valuable therapeutic effect when used in combination with antibiotic treatment. Therefore, the potential use of optimized therapies to enhance chemotherapeutic efficacy by increasing immune responses should be more carefully designed using appropriate preclinical animal models. Although an improved understanding of interactions between the host and pathogen in the context of MAC disease could reveal additional host protective immune responses, strategies for improving the immune defense response are required to protect against pathogenesis and modulate pathologic inflammation by reducing or reversing mycobacterial-induced lung damage by therapeutic means. Collectively, our results highlight the value of better understanding the mechanisms underlying vaccination strategies according to the status of MAC infection and vaccination intentions. Due to this significantly emerging health threat and the underestimated true incidence of MAC-PD, novel adjunctive therapeutic approaches, including the development of vaccines for preventing MAC infection, should be further investigated to improve the prevention, treatment outcomes, treatment duration, and therapeutic efficacy based on understanding the reciprocal interactions between current antibiotics for the treatment of MAC infection and host immune cells.

## Supplementary Material

Supplemental Material

## Data Availability

The raw data that support the findings of this study are available from the corresponding author upon reasonable request.
