# Supplementary material for: Vaccination inducing durable and robust antigen-specific Th1/Th17 immune responses contributes to prophylactic protection against Mycobacterium avium infection but is ineffective as an adjunct to antibiotic treatment in chronic disease
Source: Virulence. 2022 May 1;13(1):808–32. doi: 10.1080/21505594.2022.2068489 (PMC9067471; doi:10.1080/21505594.2022.2068489)
Supplement: Supplemental Material [file KVIR_A_2068489_SM3738.zip › supplementary/Revised supplementary table_KVIR-2022-0001R2.docx]

Supplementary Table 1. The preventative vaccination and therapeutic vaccination challenge in BALB/c mice

| **The preventative vaccination** | | | | |
| --- | --- | --- | --- | --- |
| Group | Condition for immunization (per mouse) | | | |
|  | Compound | Administration route | Volume | Immunization times |
| GLA-SE | 5 μg GLA-SE | intramuscular | 100 μl | 3 times at 3-week intervals |
| CFA+GLA-SE | 5 μg CFA and 5 μg GLA-SE | intramuscular | 100 μl | 3 times at 3-week intervals |
| CFA+GLA-SE/CDG | 5 μg CFA , 5 μg GLA-SE, and 5 μg CDG | intramuscular | 100 μl | 3 times at 3-week intervals |
| **The therapeutic vaccination after 8 weeks infection** | | | | |
| Group | Condition for immunization (per mouse) | | | |
|  | Compound | Administration route | Volume | Immunization times |
| Infection only | X | intramuscular | X | X |
| CFA+GLA-SE/CDG | 5 μg CFA5 μg GLA-SE, and 5 μg CDG | intramuscular | 100 μl | 3 times at 3-week intervals |
| CLR | 2 mg (100mg/kg) | Oral | 200 μl | Daily for 4 weeks |
| CLR+CFA+GLA-SE/CDG | 2 mg CLR, 5 μg CFA, 5 μg GLA-SE, and 5 μg CDG | Oral (CLR) Intramuscular (CFA+GLA-SE/CDG) | 200 μl (CLR)  100 μl (CFA+GLA-SE/CDG) | Daily for 4 weeks (CLR) 3 times at 3-week intervals (CFA+GLA-SE/CDG) |
| Abbreviation: GLA-SE, glucopyranosyl lipid A adjuvant formulated in a stable oil-in-water emulsion; CFA, culture filtrate antigen; GLA-SE/CDG, GLA-SE plus cyclic-di-GMP; CLR, clarithromycin | | | | |

Supplementary Table 2. List of fluorescently conjugated antibodies used in this study

| **Antibodies** | **Dilution** | **Source** | **Identifier** |
| --- | --- | --- | --- |
| Unconjugated rat monoclonal anti-mouse CD16/32 (clone 93) | 1/400 | BioLegend | Cat. No. 101320 |
|  |  |  | RRID:AB_1574975 |
| **Surface staining** | | | |
| LIVE/DEAD^TM^ Fixable Near-IR dead cell stain kit | 1/500 | ThermoFisher Scientific | Cat. No. L34976 |
| Violet 450 rat monoclonal anti-mouse CD44 (clone IM7) | 1/300 | BD Biosciences | Cat. No. 560451 RRID:AB_1645273 |
| PerCP-Cy^TM^5.5 rat monoclonal anti-mouse CD4 (clone RM4-5) | 1/300 | BD Biosciences | Cat. No. 550954 |
|  |  |  | RRID:AB_393977 |
| Brilliant Violet 605^TM^ rat monoclonal anti-mouse CD90.2 (Thy-1.2) (clone 53-2.1) | 1/300 | BioLegend | Cat. No. 140318 |
|  |  |  | RRID:AB_2650924 |
| Brilliant Violet 785 ^TM^ rat monoclonal anti-mouse CD8a (clone 53-6.7) | 1/300 | BioLegend | Cat. No. 100750 |
|  |  |  | RRID:AB_252610 |
| Alexa Fluor 700 rat monoclonal anti-mouse CD62L (clone MEL-14) | 1/300 | BioLegend | Cat. No. 104426 |
|  |  |  | RRID:AB_493719 |
| PE rat monoclonal anti-human/mouse CD44 (clone IM7) | 1/300 | Invitrogen | Cat. No. 12-0441-82 RRID:AB_465664 |
| PE/Dazzle^TM^ 594 armenian hamster monoclonal anti-mouse CD11c (clone N418) | 1/300 | BioLegend | Cat. No. 117348 RRID:AB_2563655 |
| FITC rat monoclonal anti-mouse CD40 (clone 3/23) | 1/300 | BioLegend | Cat. No. 124607 RRID:AB_1134090 |
| PE rat monoclonal anti-human/mouse CD86 (B7-2) (clone GL1) | 1/300 | Invitrogen | Cat. No. 12-0862-83 RRID:AB_465769 |
| PE mouse monoclonal anti-mouse MHC Class I (H-2Kd/H-2Dd) (clone 34-1-2S) | 1/300 | Invitrogen | Cat. No. 12-5998-83 RRID:AB_466123 |
| APC-Cy7 rat monoclonal anti-mouse MHC Class II(clone M5/114.15.2) | 1/300 | BioLegend | Cat. No. 107628 RRID:AB_2069377 |
| **Intracellular staining** | | | |
| Alexa Fluor 488 rat monoclonal anti- mouse IL-17A (clone TC11-18H10) | 1/200 | BD Biosciences | Cat. No. 560220 |
|  |  |  | RRID:AB_1645194 |
| PE rat monoclonal anti-mouse IFN-γ (clone XMG1.2) | 1/200 | BD Biosciences | Cat. No. 554412 |
|  |  |  | RRID:AB_395376 |
| PE-Cy7 rat monoclonal anti-mouse IL-2 antibody (clone JES6-5H4) | 1/200 | BD Biosciences | Cat. No. 560538 |
|  |  |  | RRID:AB_1727545 |
| APC rat monoclonal anti-mouse TNF-α (MP6-XT22) | 1/200 | BD Biosciences | Cat. No. 554420 |
|  |  |  | RRID:AB_398553 |
| APC rat monoclonal anti-mouse ROR gamma (t) antibody (clone AFKJS-9) | 1/100 | Invitrogen | Cat. No. 17-6988-82 |
|  |  |  | RRID:AB_10609207 |
| PE/Dazzle^TM^ 594 mouse monoclonal anti-mouse T-bet (clone 4B10) | 1/100 | BioLegend | Cat. No. 644828 |
|  |  |  | RRID:AB_2565677 |

Supplementary Table 3. The ELISA conditions used in this study

| Antibodies (standard) | Top concentration (pg/ml)^a^ | Source | Sample dilution^b^ |
| --- | --- | --- | --- |
| IFN-γ | 2000 | Invitrogen | 1:5 |
| TNF-α | 1000 | Invitrogen | 1:10 |
| IL-17A | 1000 | Invitrogen | 1:1 |
| IL-2 | 2000 | Invitrogen | 1:2 |
| IL-10 | 2000 | Biolegend | 1:2 |
| ^a^ Use the top standard solution of each antibody to produce dilution series within assay diluent. The zero standard (0 pg/ml) was the assay diluent. ^b^ Samples were diluted in assay diluent. | | | |

Supplementary Table 4. A summary of bacterial reduction and T cell producing cytokines profile via the preventative and therapeutic vaccine challenges

| **The preventative vaccine** | **Reduction** | **Production of T cell cytokines** | | | | | **Major T cell type** |
| --- | --- | --- | --- | --- | --- | --- | --- |
|  | **(% to GLA-SE)** | **INF-γ** | **IL-17A** | **IL-2** | **TNF-α** | **IL-10** |  |
| GLA-SE | - | - | - | - | + | + | - |
| CFA+GLA-SE | 5.1% | - | + | + | + | + | CD4^+^CD44^+^IFN-γ^+^IL-2^+^ CD4^+^CD44^+^IFN-γ^+^TNF-α^+^ |
| CFA+GLA-SE/CDG | 17.7% | + | + | + | + | + | CD4^+^CD44^+^IFN-γ^+^IL-2^+^ CD4^+^CD44^+^IFN-γ^+^IL-17A+ CD4^+^CD44^+^IFN-γ^+^TNF-α^+^ |
| **The therapeutic vaccine** | **Reduction** | **Production of T cell cytokines** | | | | | **Major T cell type** |
|  | **(% to control)** | **INF-γ** | **IL-17A** | **IL-2** | **TNF-α** | **IL-10** |  |
| Infection control | - | + | + | - | + | + | - |
| CFA+GLA-SE/CDG | 5.2% | + | + | + | + | + | CD4^+^CD44^+^IFN-γ^+^IL-17A+ CD4^+^CD44^+^IFN-γ^+^TNF-α^+^ |
| CLR | 26.1% | + | + | + | + | + | CD4^+^CD44^+^IFN-γ^+^IL-2^+^ CD4^+^CD44^+^IFN-γ^+^IL-17A+ CD4^+^CD44^+^IFN-γ^+^TNF-α^+^ |
| CLR+CFA+GLA-SE/CDG | 25.8% | + | + | + | + | + | CD4^+^CD44^+^IFN-γ^+^IL-2^+^ CD4^+^CD44^+^IFN-γ^+^IL-17A+ CD4^+^CD44^+^IFN-γ^+^TNF-α^+^ |
| The bacterial reduction, the production of cytokines and the major T cell type of indicated groups were summarized. The cytokines and the T cell types in response to 10 μg/ml CFA stimulation were shown based on ELISA and flow cytometry analyses. No response, (-); cytokine secretion, (+).  Abbreviation: GLA-SE, glucopyranosyl lipid A adjuvant formulated in a stable oil-in-water emulsion; CFA, culture filtrate antigen; GLA-SE/CDG, GLA-SE plus cyclic-di-GMP; CLR, clarithromycin | | | | | | | |
