# Supplementary material for: Vaccination inducing durable and robust antigen-specific Th1/Th17 immune responses contributes to prophylactic protection against Mycobacterium avium infection but is ineffective as an adjunct to antibiotic treatment in chronic disease
Source: Virulence. 2022 May 1;13(1):808–32. doi: 10.1080/21505594.2022.2068489 (PMC9067471; doi:10.1080/21505594.2022.2068489)
Supplement: Supplemental Material [file KVIR_A_2068489_SM3738.zip › supplementary/Supplemental information_Clean copy.docx]

**Supplementary Figure 1.** Pathogenesis in two different murine models of chronic progressive Mav-PI (C57BL/6 and BALB/c). (A) Scheme of the *in vivo* experiment. The bacterial burdens (B) in the left lung lobes and whole spleens of five mice of each strain were assessed by counting viable bacterial colonies grown on 7H10-OADC agar plates, and the results are presented as a scatter plot with bars. (C) Photomicrographs at 10× magnification with H&E staining (scale bar = 2 mm) of the right superior lobe of infected lung tissues of all mice are displayed. (D) Quantitative analysis of the inflamed areas in the H&E-stained lung tissues. The sizes and percentages of the lesions in (C) and the data are presented as a scatter plot with bars. The statistical significance in (A), (B) and (D) was determined by the unpaired *t test*, and the results are presented as the mean values along with the S.Ds. **p* < 0.05, ***p* < 0.01. The representative results are shown from a single *in vivo* experiment. a.i., aerosol infection.

**Supplementary Figure 2.** The gating strategy used for the identification of CFA-specific multifunctional CD4^+^ T cell populations upon *ex vivo* stimulation by GolgiPlug and GolgiStop with or without 10 μg/ml CFA at 37°C for 9 h.

**Supplementary Figure 3.** Qualitative and quantitative analyses of Mav CFA-specific multifunctional CD4^+^ T cells in the spleen after CFA+GLA-SE or CFA+GLA-SE/CDG immunization. At four weeks after the last immunization, mice were sacrificed, and spleen single cells harvested from each group (n = 4) were stimulated by GolgiPlug and GolgiStop with or without 10 μg/ml CFA at 37°C for 9 h. The frequencies of Mav CFA-specific IFN-γ^+^IL-2^+^-, IFN-γ^+^IL-17A^+^- and IFN-γ^+^TNF-α^+^-expressing CD4^+^CD44^+^CD62L^-^ T cells were assessed after staining of intracellular cytokines and are presented as (A) pseudocolor dot plots and (B) summary bar graphs. (C) The percentages of total CD4^+^CD44^+^CD62L^-^ T cells with differential production of IFN-γ, IL-2, IL-17A and TNF-α in response to CFA stimulation among spleen single cells were determined among groups and are presented as bar graphs. (D) The values of the proportions of quadruple- (4+, crimson), triple- (3+, orange), double- (2+, yellow), and single-function (1+, light gray) CD4^+^CD44^+^CD62L^-^ T cells expressing IFN-γ, IL-2, IL-17A and TNF-α in each immunized group and naïve group are illustrated as pie charts. Statistically significant differences among all groups in (B) and (C) were determined by one-way ANOVA with Tukey’s multiple comparison test, and the results are presented as the mean values along with the S.Ds. **p* < 0.05, ***p* < 0.01, ****p* < 0.001, *****p* < 0.0001 and *n.s.:* not significant. The asterisks in (C) represent significant differences between groups: black, control group vs. CFA+GLA-SE/CDG group; red, CFA+GLA-SE group vs. CFA+GLA-SE/CDG group. The representative results are shown from a single *in vivo* experiment. CFA, culture filtrate antigen; Control, GLA-SE immunization alone; GLA-SE, glucopyranosyl lipid A adjuvant formulated in a stable oil-in-water emulsion; GLA-SE/CDG, GLA-SE plus cyclic-di-GMP.

**Supplementary Figure 4.** Humoral responses elicited by subunit vaccines formulated with different adjuvants. Mouse sera from BALB/c mice in each immunized group (n = 4) were evaluated for CFA-specific (A) total IgG, (B) IgG1, (C) IgG2a and (D) IgG2b levels, and the results are presented as a scatter plot with bars. Statistically significant differences were determined by one-way ANOVA with Tukey’s multiple comparison test, and the results are presented as the mean values along with the S.Ds. **p* < 0.05, ****p* < 0.001, *****p* < 0.0001 and *n.s.:* not significant. The representative results are shown from a single *in vivo* experiment. Control, GLA-SE immunization alone; CFA, culture filtrate antigen; GLA-SE, glucopyranosyl lipid A adjuvant formulated in a stable oil-in-water emulsion; GLA-SE/CDG, GLA-SE plus cyclic-di-GMP.

**Supplementary Figure 5.** Phenotypical maturation and T cell polarization induced by GLA and CDG stimulated DCs. (A) Bone marrow-derived DCs were stimulated for 24 h with LPS (100 ng/ml), GLA (50 ng/ml), CDG (200 ng/ml), or GLA+CDG. Then, cells were analyzed for the expression of cell surface markers by flow cytometry. The bar graphs show the mean values along with the S.Ds. of the staining intensity of each indicated surface marker on the CD11c^+^ cells. (B) Bone marrow-derived DCs were stimulated with GLA (50 ng/ml), CDG (200 ng/ml), or both. Then, OVA_323-339_ peptide (1 μg/ml)-pulsed DCs were cocultured with CD4^+^ T cells isolated from spleen of OT-II mice. After 3 days of coculture, T cell polarization were assessed by flow cytometry. Statistically significant differences were determined by one-way ANOVA with Tukey’s multiple comparison test. Data in graphs are expressed as mean value along with the S.Ds. **p* < 0.05, ***p* < 0.01, ****p* < 0.001, *****p* < 0.0001 and *n.s.:* not significant. DCs, dendritic cells; GLA, glucopyranosyl lipid A; CDG, cyclic-di-GMP.

**Supplementary Figure 6.** Quantitative and qualitative analyses of the long-lasting induction of Mav CFA-specific multifunctional CD4^+^ T cells after CFA+GLA-SE or CFA+GLA-SE/CDG immunization in the spleen in a murine model of chronic progressive Mav-PI at 10 weeks post-infection. At 10 weeks post-infection, mice were sacrificed, and spleen cells harvested from each immunized group (n = 6) and naïve group (n = 4) were stimulated by GolgiPlug and GolgiStop with or without 10 μg/ml CFA at 37°C for 9 h. The frequencies of Mav CFA-specific IFN-γ^+^IL-2^+^-, IFN-γ^+^IL-17A^+^- and IFN-γ^+^TNF-α^+^-expressing CD4^+^CD44^+^CD62L^-^ T cells were assessed after staining of intracellular cytokines and are presented as (A) pseudocolor dot plots and (B) summary bar graphs. (C) The percentages of total CD4^+^CD44^+^CD62L^-^ T cells with differential production of IFN-γ, IL-2, IL-17A and TNF-α in response to CFA stimulation among spleen single cells were determined among groups and are presented as bar graphs. (D) The values of the proportions of quadruple- (4+, crimson), triple- (3+, orange), double- (2+, yellow), and single-function (1+, light gray) CD4^+^CD44^+^CD62L^-^ T cells expressing IFN-γ, IL-2, IL-17A and TNF-α in each immunized group and naïve group are illustrated as pie charts. Statistically significant differences among all groups in (B) and (C) were determined by one-way ANOVA with Tukey’s multiple comparison test, and the results are presented as the mean values along with the S.Ds. **p* < 0.05, ***p* < 0.01, ****p* < 0.001, *****p* < 0.0001 and *n.s.:* not significant. The representative results are shown from a single *in vivo* experiment. The asterisks in (C) represent significant differences between groups: black, control group vs. CFA+GLA-SE/CDG group; red, CFA+GLA-SE group vs. CFA+GLA-SE/CDG group. CFA, culture filtrate antigen; Control, GLA-SE immunization alone; GLA-SE, glucopyranosyl lipid A adjuvant formulated in a stable oil-in-water emulsion; GLA-SE/CDG, GLA-SE plus cyclic-di-GMP.

**Supplementary Figure 7.** Humoral responses elicited by subunit vaccines containing different adjuvants in a murine model of chronic progressive Mav-PI at 10 weeks post-infection. Mouse sera from BALB/c mice in each immunized group (n = 6) and naïve group (n = 4) were evaluated for CFA-specific (A) total IgG, (B) IgG1, (C) IgG2a and (D) IgG2b levels, and the results are presented as a scatter plot with bars. Statistically significant differences were determined by one-way ANOVA with Tukey’s multiple comparison test, and the results are presented as the mean values along with the S.Ds. ***p* < 0.01, ****p* < 0.001, *****p* < 0.0001 and *n.s.:* not significant. The representative results are shown from a single *in vivo* experiment. Control, GLA-SE immunization alone; CFA, culture filtrate antigen; GLA-SE, glucopyranosyl lipid A adjuvant formulated in a stable oil-in-water emulsion; GLA-SE/CDG, GLA-SE plus cyclic-di-GMP.

**Supplementary Figure 8.** Anti-Mav activities of IFN-γ and IL-17A in Mav-infected primary macrophages. Mav-infected BMDMs were treated with 20 ng/ml IFN-γ, 20 ng/ml IL-17A, 10 ng/ml of both, or 20 ng/ml of both. After 3 days of incubation, Mav CFUs were assessed by plating serial dilutions of cell lysates on 7H10-OADC agar plates. Data from a representative experiment is presented as a scatter plot with bars. Statistically significant differences were determined by one-way ANOVA with Tukey’s multiple comparison test, and each dot of triplicate wells, with four spots applied per well, is presented as the mean values along with the S.Ds. ***p* < 0.01, *****p* < 0.0001 and *n.s.:* not significant. Pre-Tx, Pre-treatment; Control, untreated infection control.

**Supplementary Figure 9.** Qualitative and quantitative analyses of Mav CFA-specific multifunctional spleen CD4^+^ T cells after treatment with CLR to boost CFA+GLA-SE/CDG immunization in a murine model of chronic progressive Mav-PI at 19 weeks post-infection. At 19 weeks post-infection, mice were sacrificed, and spleen cells of each treatment group (n = 5) and infection control group (n = 4) were stimulated by GolgiPlug and GolgiStop with or without 10 μg/ml CFA at 37°C for 9 h. The frequencies of Mav CFA-specific IFN-γ^+^IL-2^+^-, IFN-γ^+^IL-17A^+^- and IFN-γ^+^TNF-α^+^-expressing CD4^+^CD44^+^CD62L^-^ T cells were assessed after staining of intracellular cytokines and are presented as (A) pseudocolor dot plots and (B) summary bar graphs. (C) The percentages of total CD4^+^CD44^+^CD62L^-^ T cells with differential production of IFN-γ, IL-2, IL-17A and TNF-α in response to CFA stimulation among spleen single cells were determined among groups and are presented as bar graphs. (D) The values of the proportions of quadruple- (4+, crimson), triple- (3+, orange), double- (2+, yellow), and single-function (1+, light gray) CD4^+^CD44^+^CD62L^-^ T cells expressing IFN-γ, IL-2, IL-17A and TNF-α in each infected, immunized, and treated group are illustrated as pie charts. Statistically significant differences among all groups in (B) and (C) were determined by one-way ANOVA with Tukey’s multiple comparison test, and the results are presented as the mean values along with the S.Ds. **p* < 0.05, ***p* < 0.01, ****p* < 0.001, *****p* < 0.0001 and *n.s.:* not significant. The asterisks in (C) represent significant differences between groups: black, control group vs. CLR group or CLR+CFA+GLA-SE/CDG group; red, CLR group vs. CLR+CFA+GLA-SE/CDG group. The representative results are shown from a single *in vivo* experiment. CFA, culture filtrate antigen; Control, untreated infection control; GLA-SE/CDG, glucopyranosyl lipid A adjuvant formulated in a stable oil-in-water emulsion plus cyclic-di-GMP; CLR, clarithromycin.

**Supplementary Figure 10.** Humoral responses regulated by antibiotic treatment with subunit vaccines containing different adjuvants in a murine model of chronic progressive Mav-PI at 19 weeks post-infection. Mouse sera from BALB/c mice in each immunized and treated group (n = 5) and untreated infection control group (n = 4) were evaluated for CFA-specific (A) total IgG, (B) IgG1, (C) IgG2a and (D) IgG2b levels, and the results are presented as a scatter plot with bars. Statistically significant differences were determined by one-way ANOVA with Tukey’s multiple comparison test, and the results are presented as the mean values along with the S.Ds. **p* < 0.05 and *n.s.:* not significant The representative results are shown from a single *in vivo* experiment. CFA, culture filtrate antigen; Control, untreated infection control; GLA-SE/CDG, glucopyranosyl lipid A adjuvant formulated in a stable oil-in-water emulsion plus cyclic-di-GMP; CLR, clarithromycin.

**Supplementary Figure 11.** Correlation analysis for the protection level elicited by vaccine-induced immune responses in preventative and therapeutic vaccination. The linear relationship between the bacterial burden (CFUs/lung) and Mav-CFA specific cytokine producing CD4^+^ T cells is illustrated as a black arrow for the (A) preventative vaccination and (B) therapeutic vaccination. Spearman’s r correlation coefficient and significance are indicated. ****p* < 0.001, and *****p* < 0.0001. The representative results are shown from a single experiment. Control, GLA-SE immunization alone (Black circle) or untreated infection control (White circle); CFA, culture filtrate antigen; GLA-SE, glucopyranosyl lipid A adjuvant formulated in a stable oil-in-water emulsion; GLA-SE/CDG, glucopyranosyl lipid A adjuvant formulated in a stable oil-in-water emulsion plus cyclic-di-GMP; CLR, clarithromycin.
