# Supplementary figures and images for: Vaccination inducing durable and robust antigen-specific Th1/Th17 immune responses contributes to prophylactic protection against Mycobacterium avium infection but is ineffective as an adjunct to antibiotic treatment in chronic disease
Source: Virulence. 2022 May 1;13(1):808–32. doi: 10.1080/21505594.2022.2068489 (PMC9067471; doi:10.1080/21505594.2022.2068489)

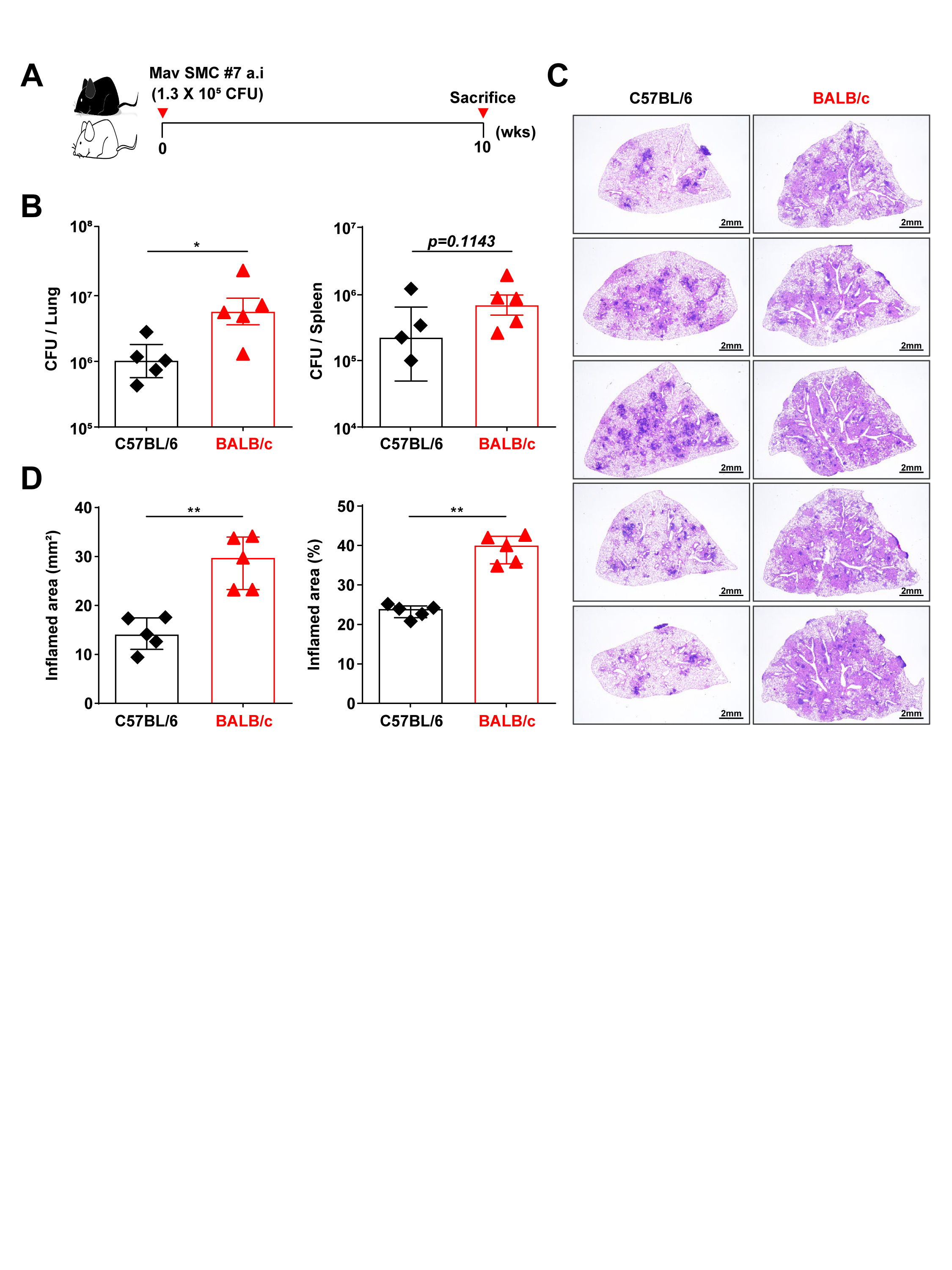

Supplement: Supplemental Material [file KVIR_A_2068489_SM3738.zip › supplementary/KVIR-2022-0001R2_Supplementary Figure 1.tif]

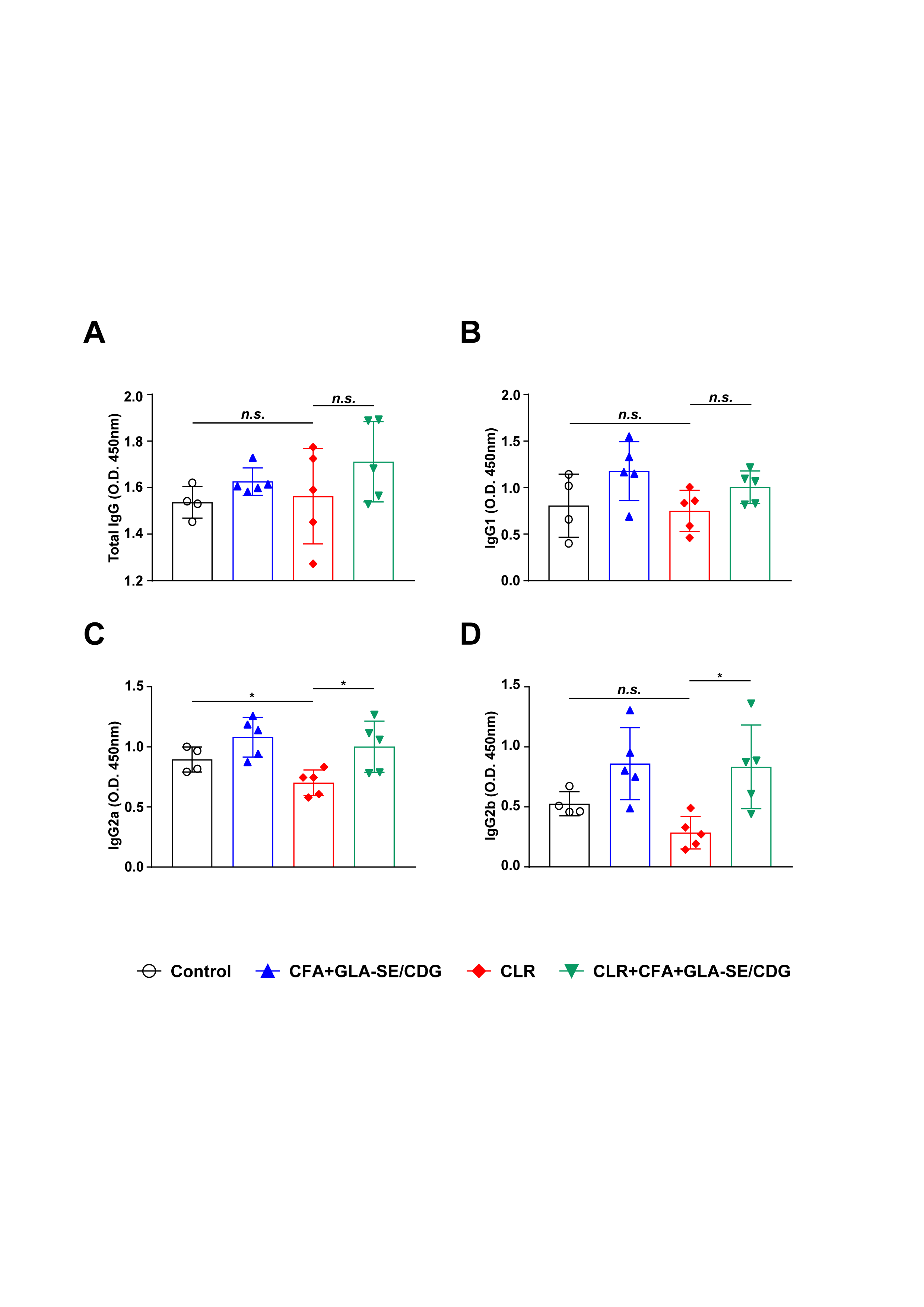

Supplement: Supplemental Material [file KVIR_A_2068489_SM3738.zip › supplementary/KVIR-2022-0001R2_Supplementary Figure 10.tif]

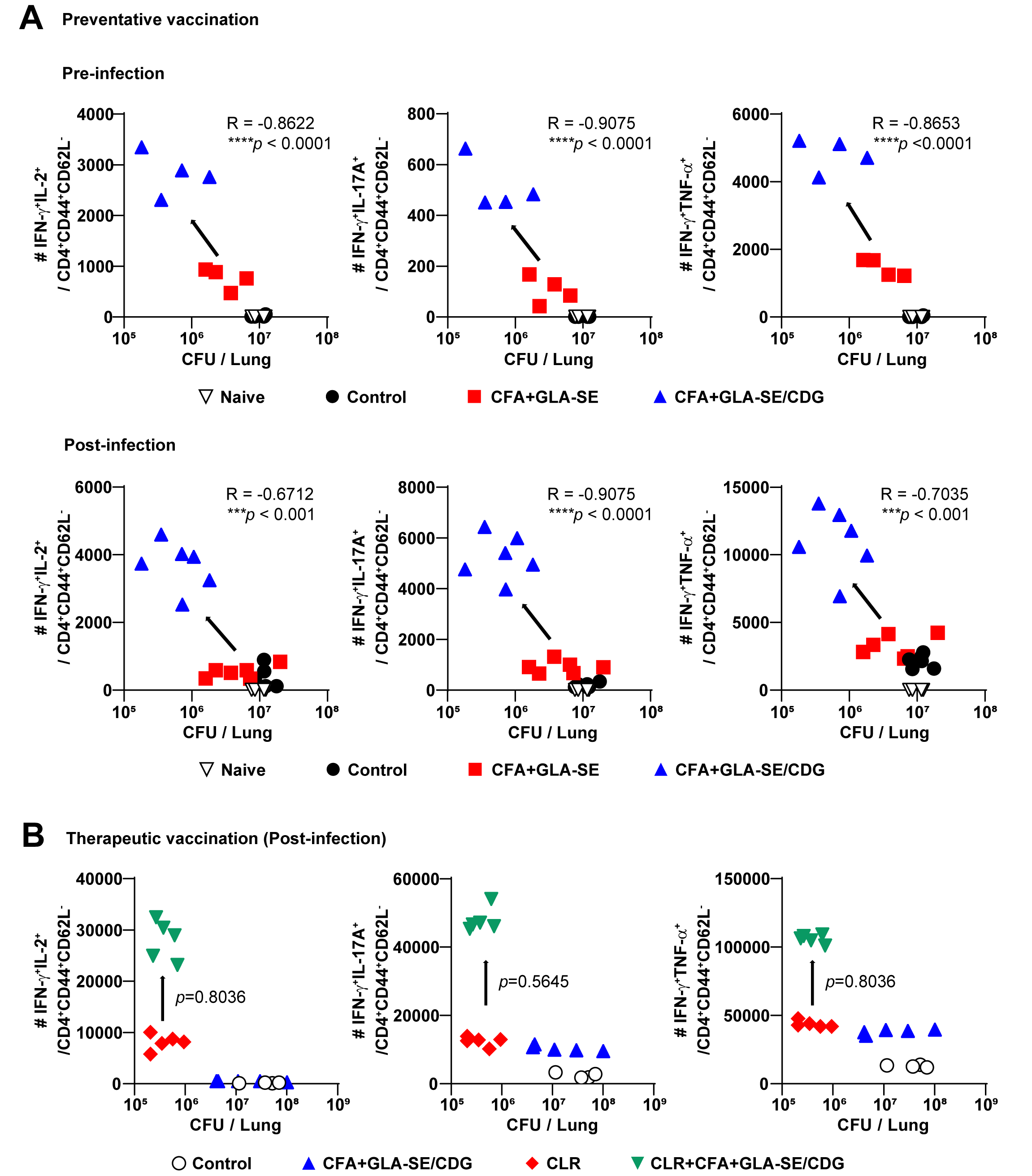

Supplement: Supplemental Material [file KVIR_A_2068489_SM3738.zip › supplementary/KVIR-2022-0001R2_Supplementary Figure 11.tif]

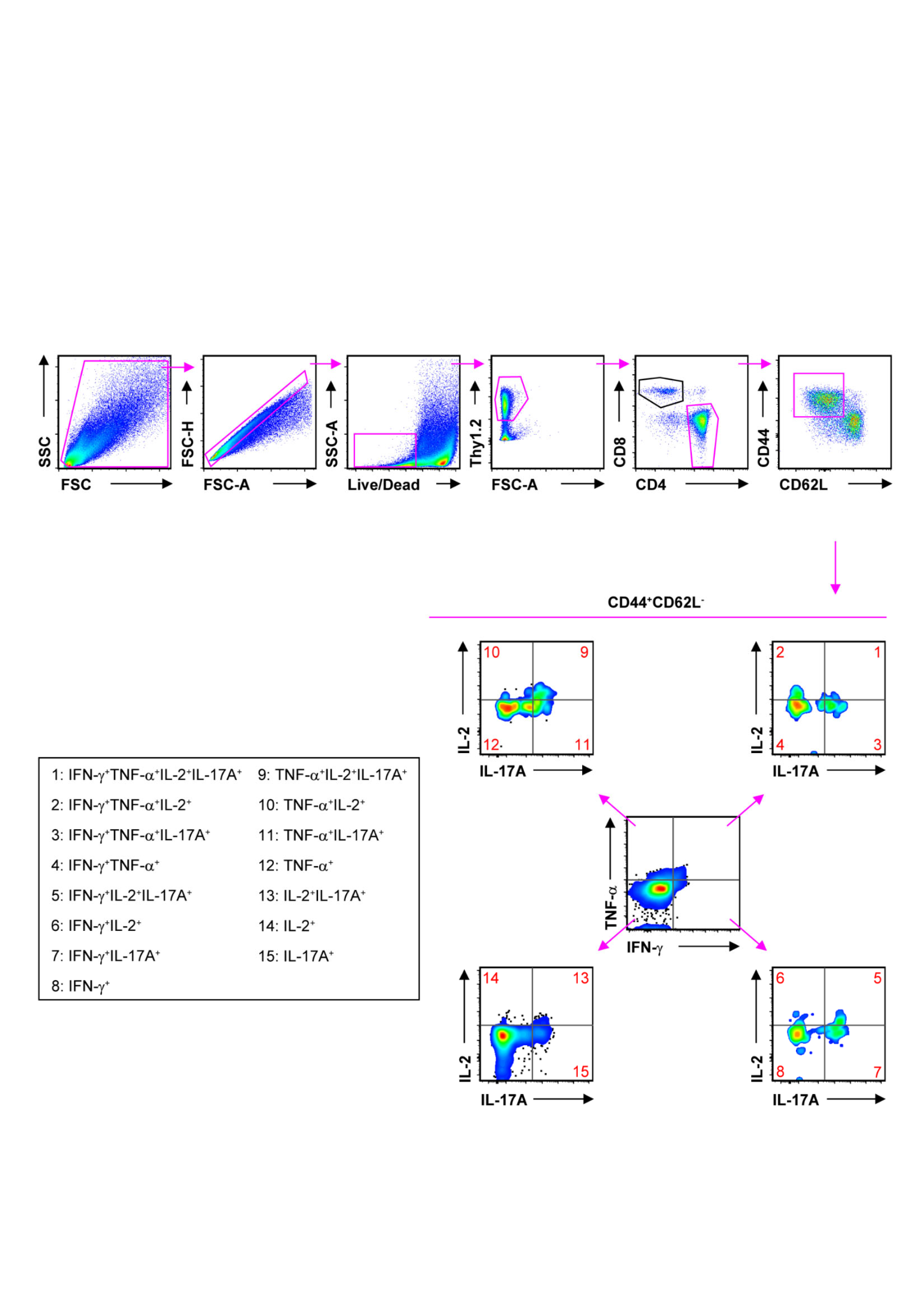

Supplement: Supplemental Material [file KVIR_A_2068489_SM3738.zip › supplementary/KVIR-2022-0001R2_Supplementary Figure 2.tif]

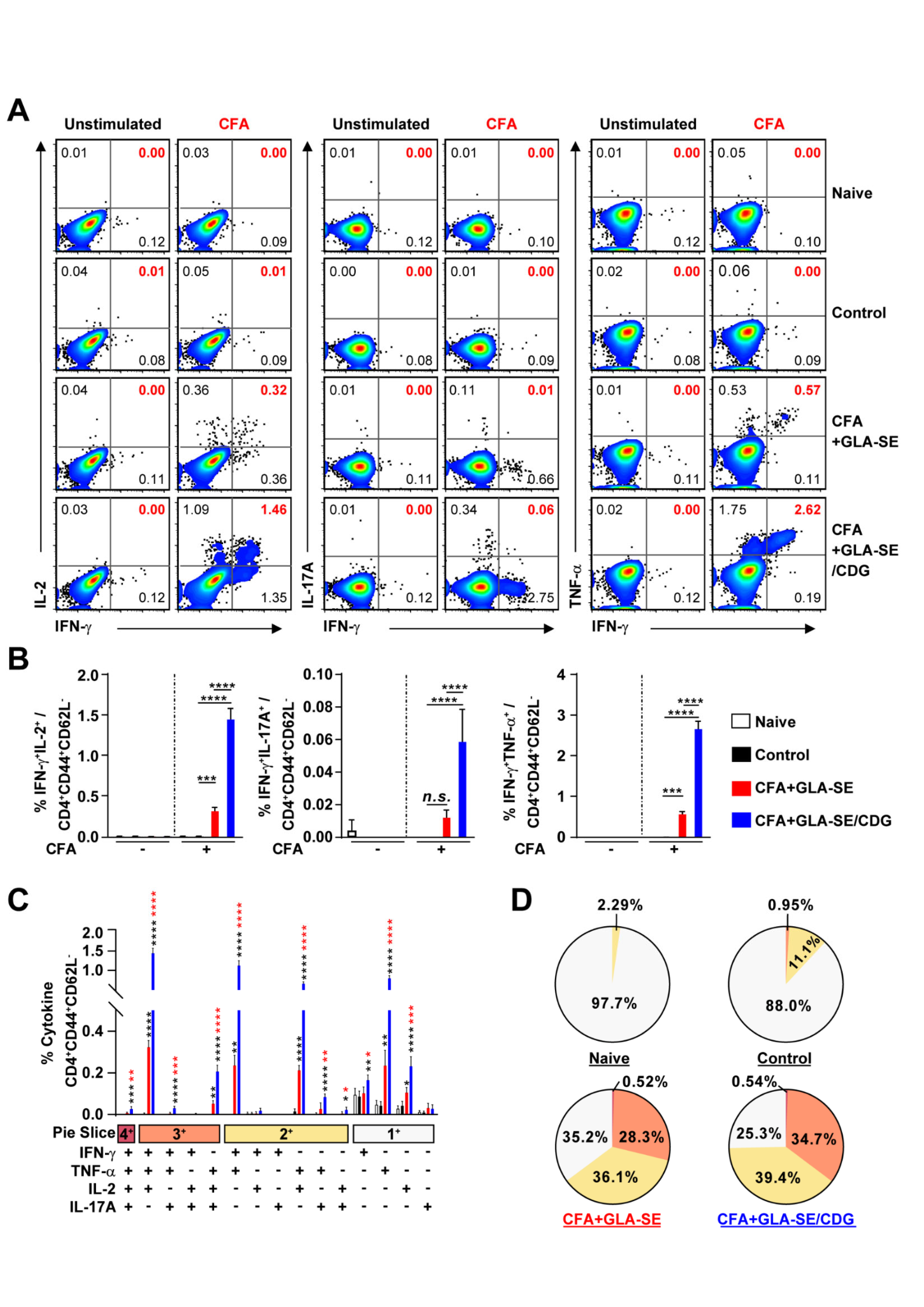

Supplement: Supplemental Material [file KVIR_A_2068489_SM3738.zip › supplementary/KVIR-2022-0001R2_Supplementary Figure 3.tif]

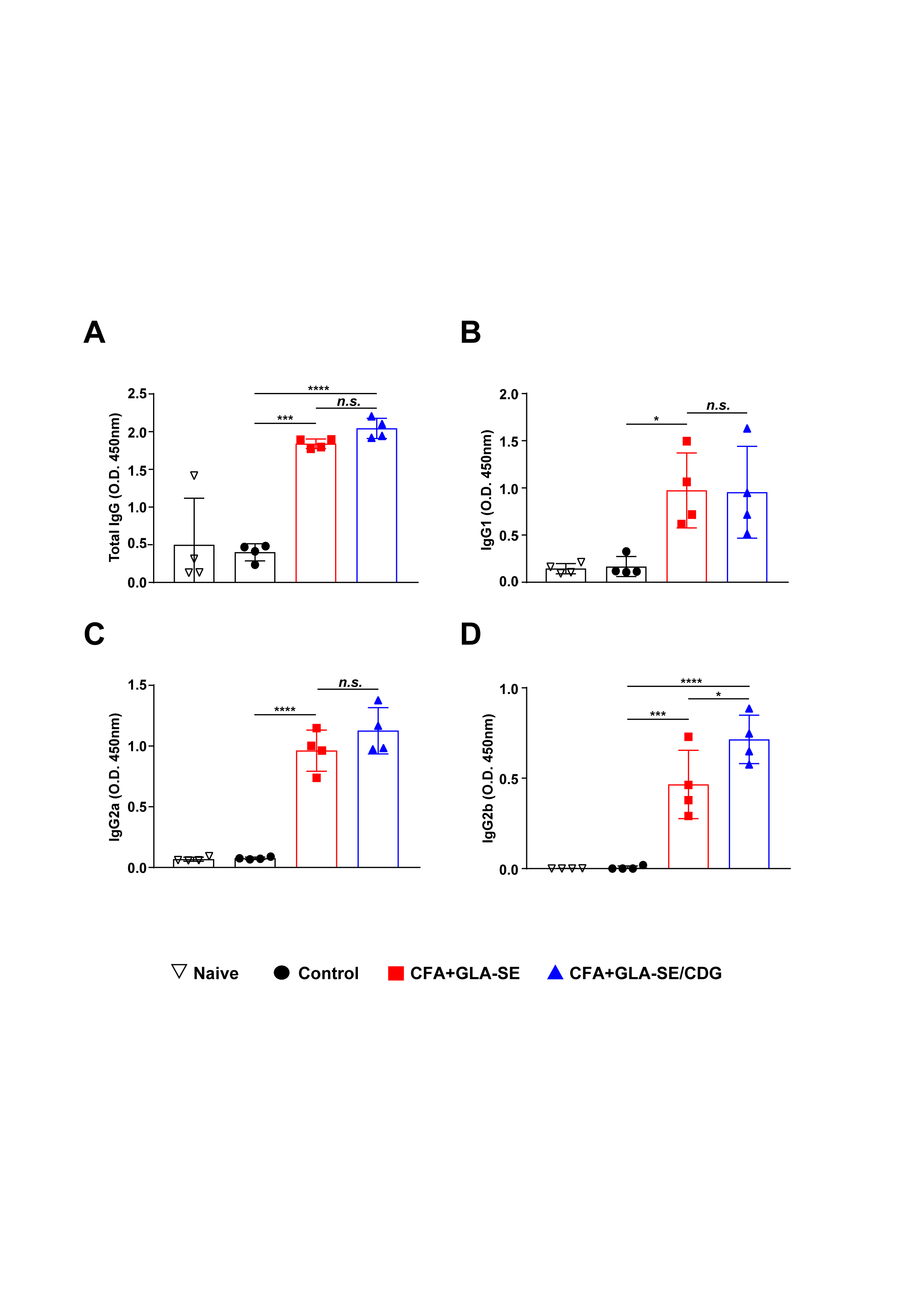

Supplement: Supplemental Material [file KVIR_A_2068489_SM3738.zip › supplementary/KVIR-2022-0001R2_Supplementary Figure 4.tif]

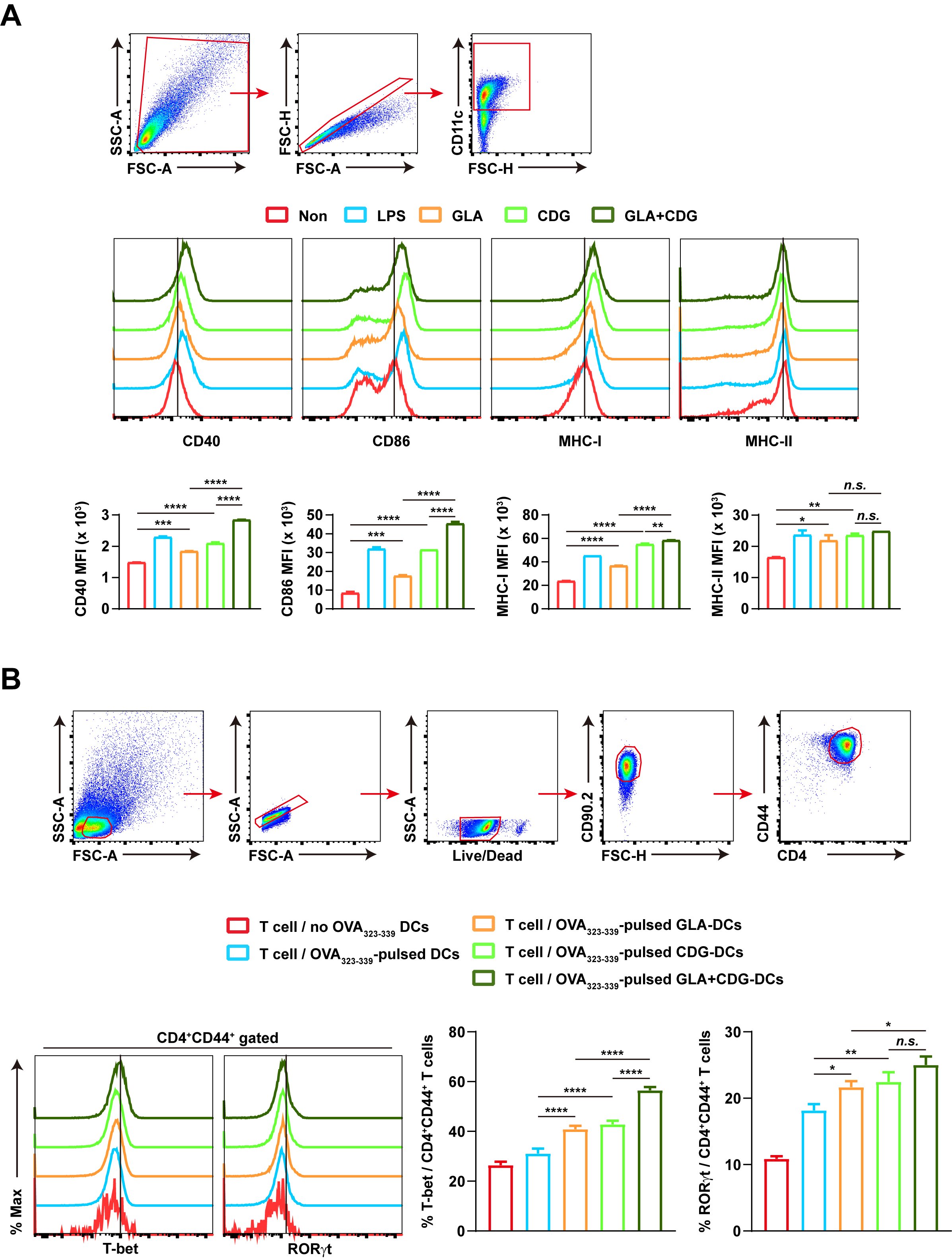

Supplement: Supplemental Material [file KVIR_A_2068489_SM3738.zip › supplementary/KVIR-2022-0001R2_Supplementary Figure 5.tif]

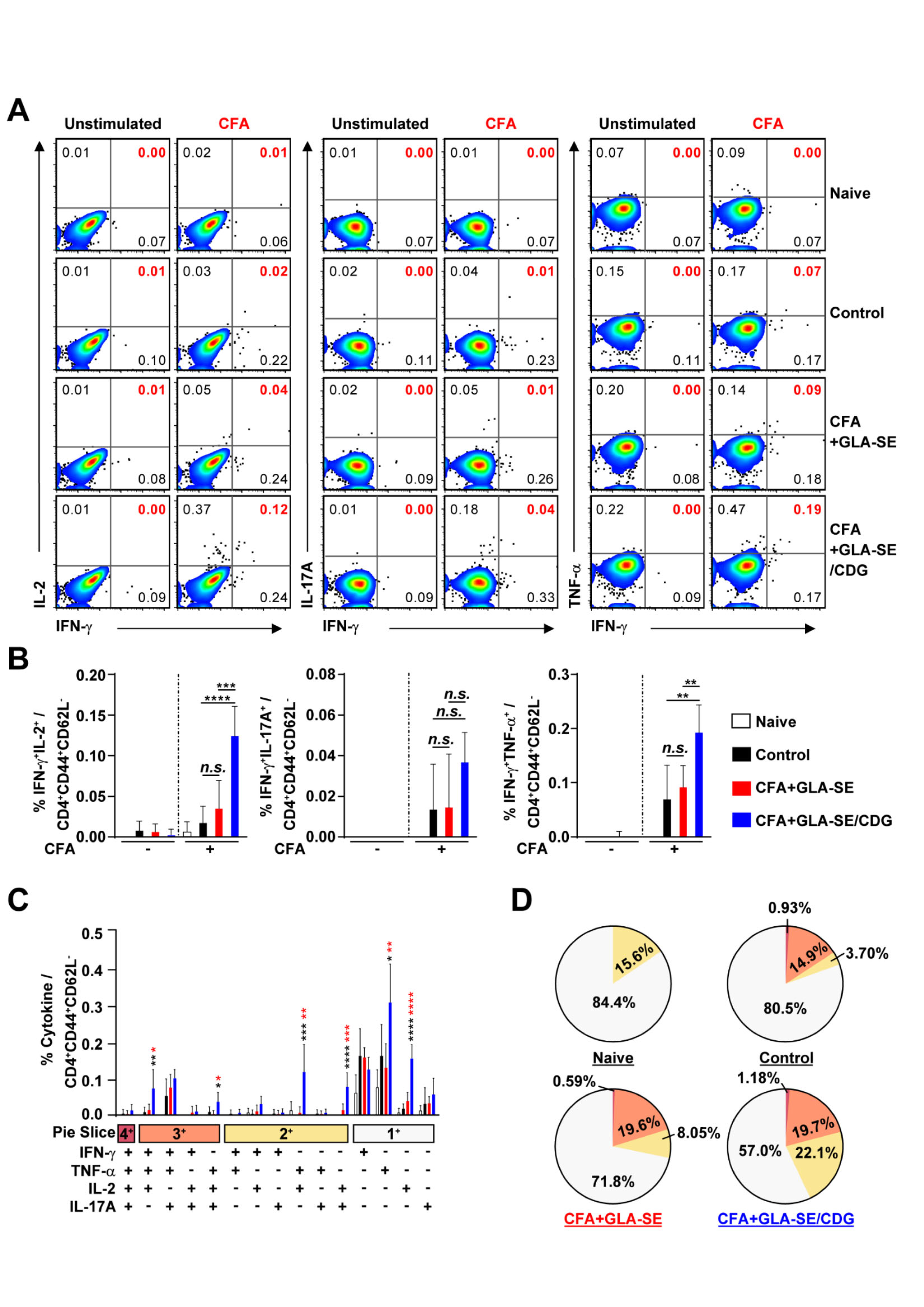

Supplement: Supplemental Material [file KVIR_A_2068489_SM3738.zip › supplementary/KVIR-2022-0001R2_Supplementary Figure 6.tif]

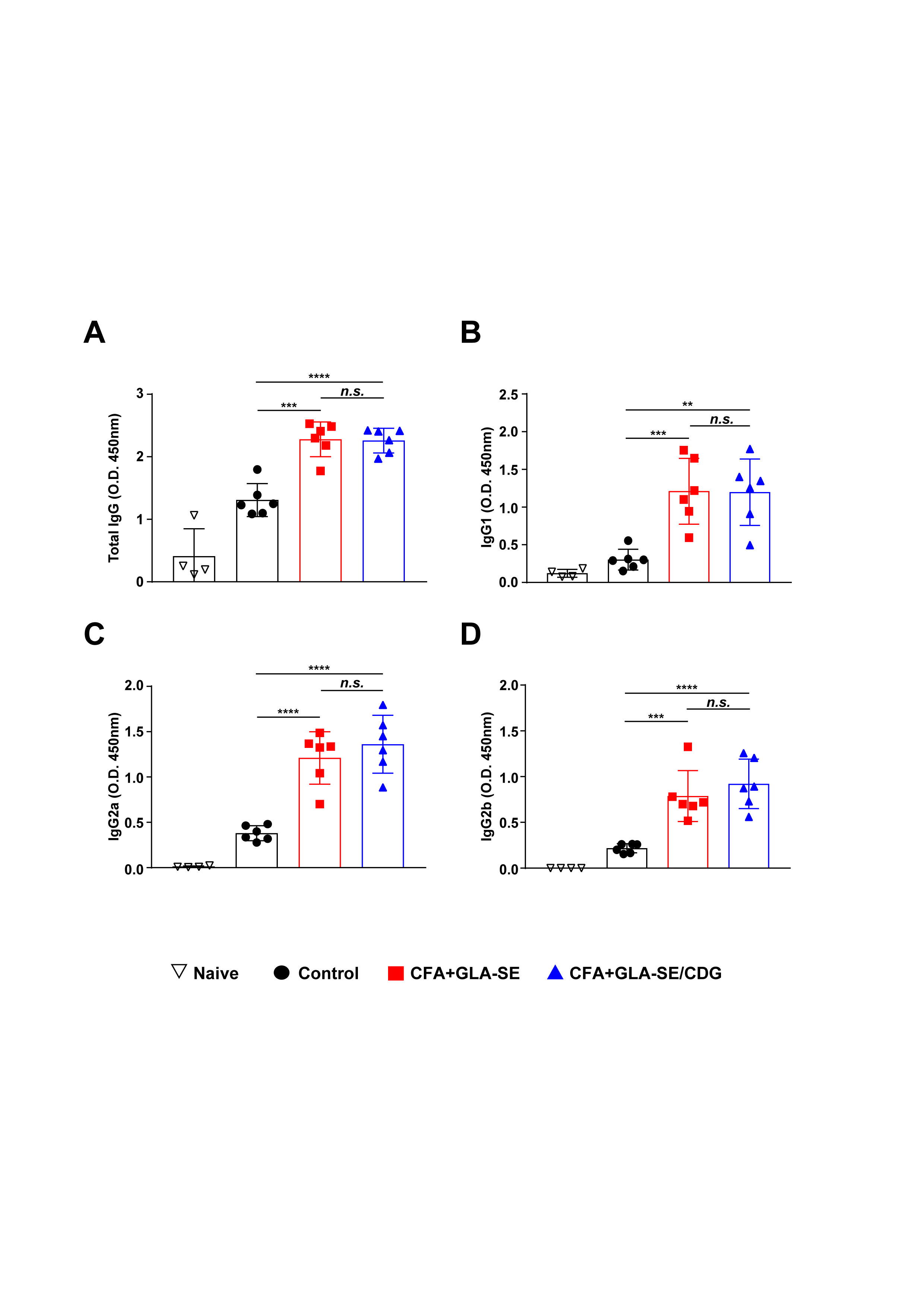

Supplement: Supplemental Material [file KVIR_A_2068489_SM3738.zip › supplementary/KVIR-2022-0001R2_Supplementary Figure 7.tif]

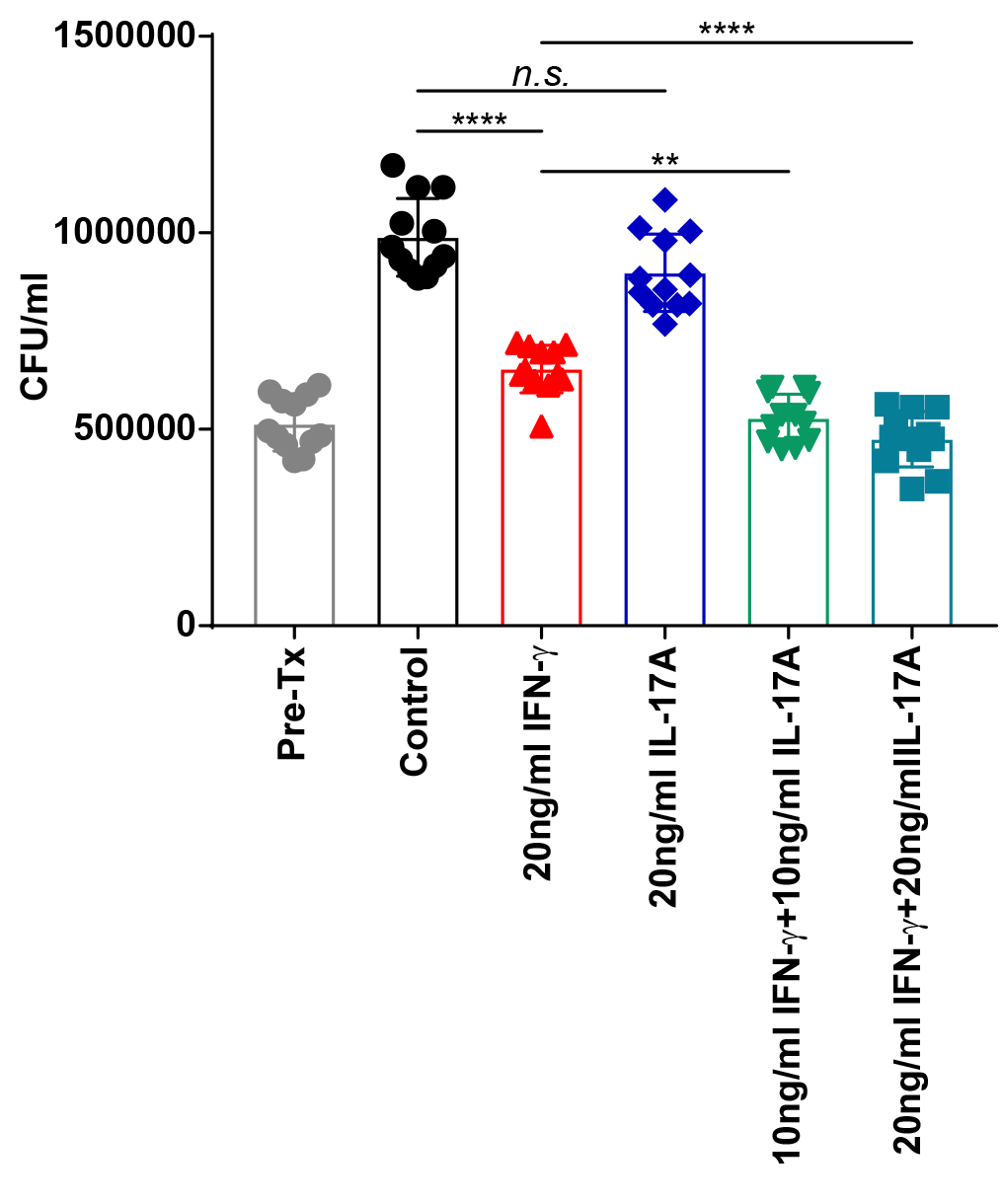

Supplement: Supplemental Material [file KVIR_A_2068489_SM3738.zip › supplementary/KVIR-2022-0001R2_Supplementary Figure 8.tif]

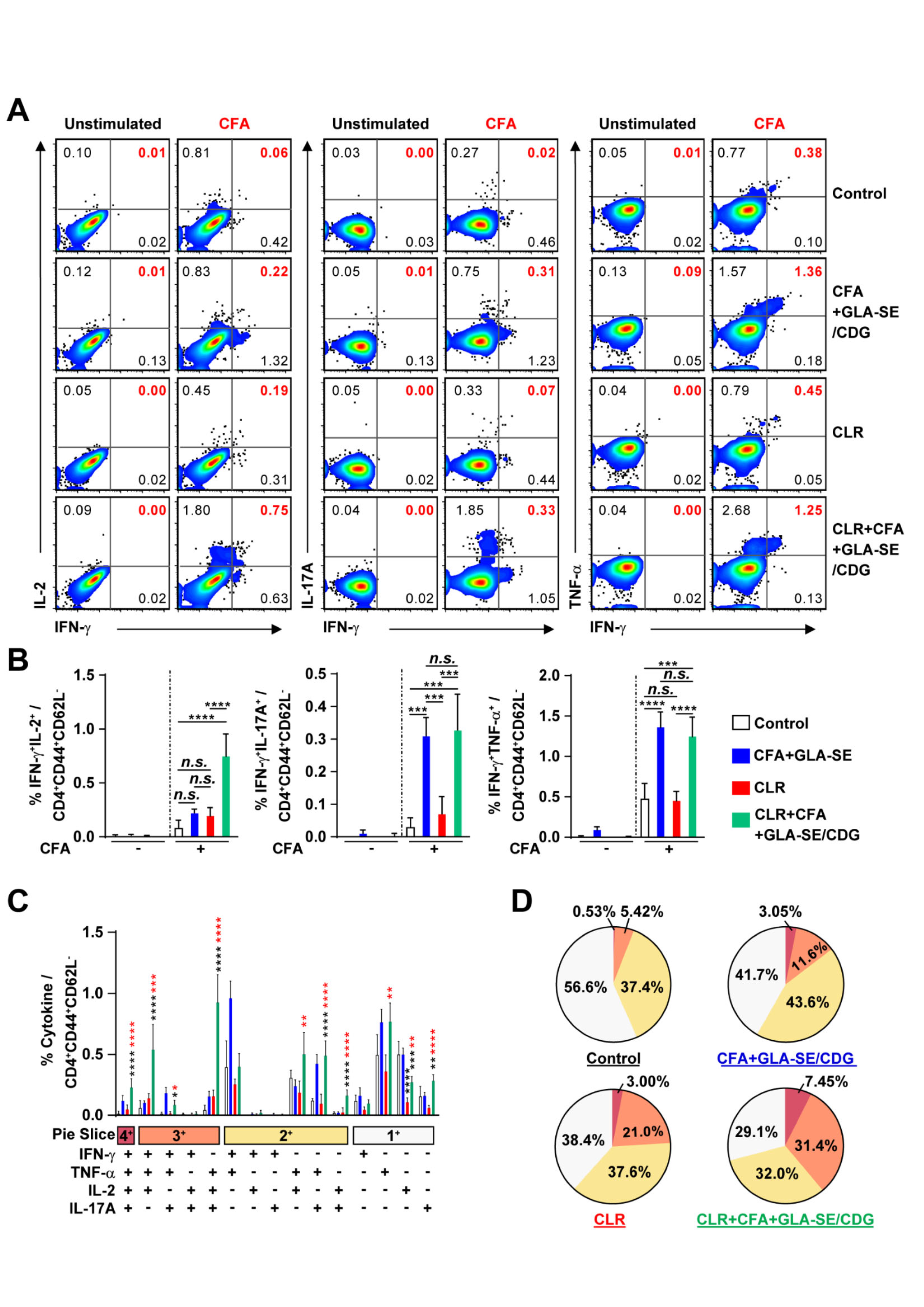

Supplement: Supplemental Material [file KVIR_A_2068489_SM3738.zip › supplementary/KVIR-2022-0001R2_Supplementary Figure 9.tif]
